# Impact and cost-effectiveness of non-governmental organizations on the HIV epidemic in Ukraine among men who have sex with men

**DOI:** 10.1097/QAD.0000000000003347

**Published:** 2022-08-10

**Authors:** Adam Trickey, Josephine G Walker, Sandra Bivegete, Nadiya Semchuk, Tetiana Saliuk, Olga Varetska, Jack Stone, Peter Vickerman

**Affiliations:** 1 Population Health Sciences, University of Bristol, Bristol, UK; 2 Alliance for Public Health, Kiev, Ukraine

**Keywords:** Harm reduction, NGO, MSM, HIV, modelling, Eastern Europe

## Abstract

**Objective:**

Non-governmental organisations (NGOs) in Ukraine have provided HIV testing, treatment, and condom distribution for men who have sex with men (MSM). HIV prevalence among MSM in Ukraine is 5.6%. We estimated the impact and cost-effectiveness of MSM-targeted NGO activities in Ukraine.

**Design:**

A mathematical model of HIV transmission among MSM was calibrated to data from Ukraine (2011-2018).

**Methods:**

The model, designed before the 2022 Russian invasion of Ukraine, evaluated the impact of 2018 status quo (SQ) coverage levels of 28% of MSM being NGO clients over 2016-2020 and 2021-2030 compared to no NGO activities over these time periods. Impact was measured in HIV incidence and infections averted. We compared the costs and disability adjusted life years [DALYs] for the SQ and a counterfactual scenario (no NGOs 2016-2020, but with NGOs thereafter) until 2030 to estimate the mean incremental cost-effectiveness ratio (ICER, cost per DALY averted).

**Results:**

Without NGO activity over 2016-2020, the HIV incidence in 2021 would have been 44% (95%CrI: 36%-59%) higher than with SQ levels of NGO activity, with 25% (21-30%) more incident infections occurring over 2016-2020. Continuing with SQ NGO coverage levels will decrease HIV incidence by 41% over 2021-2030, whereas it will increase by 79% (60-120%) with no NGOs over this period and 37% (30-51%) more HIV infections will occur. Compared to if NGO activities had ceased over 2016-2020 (but continued thereafter), the SQ scenario averts 14,918 DALYs over 2016-2030 with a mean ICER of US$600.15 per DALY averted.

**Conclusions:**

MSM-targeted NGOs in Ukraine have prevented considerable HIV infections and are highly cost-effective compared with a willingness-to-pay threshold of 50% of Ukraine’s 2018 GDP (US$1,548).

## Introduction

The Eastern Europe and Central Asia region has a fast growing HIV epidemic(1), with Ukraine having the second largest HIV burden(2). Men who have sex with men (MSM) have a high prevalence of HIV in Ukraine (5.6% in 2018(3)), however, it is difficult to estimate the overall number with HIV due to stigmatisation of MSM(4).

Most funding for HIV treatment and prevention services in Ukraine, including among MSM, goes to non-governmental organisations (NGOs)(5). NGOs targeting MSM provide HIV testing and counselling services, distribute condoms, and link HIV-diagnosed people to antiretroviral therapy (ART) at government AIDS centres. Condom use can reduce the transmission of HIV among MSM(6, 7), while ART renders HIV untransmissible if people’s viral loads are undetectable(8, 9). In our previous epidemiological analyses utilising four national integrated bio-behavioural surveys (IBBS) among MSM in Ukraine, we found that being in contact with MSM-targeted NGOs was associated with increased condom use and HIV testing, and greater likelihood of being on ART(10).

To help policymakers allocate their limited resources, information is needed on the benefits and costs of different health interventions. This has become particularly important in middle-income countries such as Ukraine because funding for NGO activities is transitioning from international funding agencies to governments(11). Although the recent invasion by Russia has radically changed Ukraine’s priorities, it is still important to show the benefit of these interventions to emphasise that services should still provide for the needs of this sub-population, and to show the potential implications of not doing so. To this end, we utilise findings from our previous epidemiological analyses(10) within a mathematical modelling framework to evaluate the impact and cost-effectiveness of MSM-targeted NGO activities on the Ukrainian HIV epidemic among MSM.

## Methods

### Model summary

We developed a dynamic, deterministic model of HIV transmission among MSM (schematic in supplementary figure 1), stratified by age (18-39 and ≥40 years), low and high sexual risk behaviour (<10 and ≥10 anal sex contacts per month), NGO status, and HIV disease progression, diagnosis, and treatment.

Individuals enter the model at age 18 as HIV-negative (susceptible) and not accessing NGOs, with a constant proportion being high-risk and the remainder low-risk. Individuals exit due to age-related or HIV-related deaths, with only the former being replaced with new entrants. Transitions occur between the low- and high-risk groups at rates that differ for ages 18-39 and ≥40 years but balance the flows between these groups. MSM become clients of NGOs at time-varying rates from 2003 and cease contact with NGOs at a constant rate. HIV-positive MSM become NGO clients at a higher rate due to NGO efforts to link HIV-positive MSM to care; our previous analyses show a higher proportion of NGO clients are HIV-positive than for non-NGO clients(10).

### HIV transmission and progression

We assume all HIV transmission is within the MSM population as few MSM (2%; IBBS) have ever injected drugs and most HIV-infected women are female sex workers(12, 13), which few MSM have sex with (last 6 months: 2%; IBBS). Uninfected MSM become infected at a rate proportional to the prevalence of HIV among MSM and the frequency of sex among low/high-risk MSM. HIV transmission is reduced through condom use, with consistency of use varying by risk level and NGO status. Differences in condom use across partnerships are averaged.

Following HIV infection, individuals progress through acute, latent, pre-AIDS, and AIDS stages of HIV disease. Transmissibility is heightened during acute infection and pre-AIDS HIV disease(14), whereas AIDS patients do not engage in sexual activity unless on ART. Individuals with AIDS die from HIV-related disease. Individuals with chronic HIV infection, pre-AIDS, or AIDS can be diagnosed and enrolled onto ART. Individuals can be lost-to-care from ART and can then re-initiate ART at the same rate as ART-naïve MSM. Individuals on ART are less infectious, and experience reduced HIV progression and mortality than individuals not on ART.

Pre-exposure prophylaxis (PrEP) is not readily available in Ukraine(15). A full model description is in the supplement.

### Model parameterisation and calibration

The model is primarily parameterised using four national IBBS among MSM implemented in 2011 (n=5,950), 2013 (n=8,101), 2015 (n=4,550), and 2018 (n=5,971)(3, 16–18). Key parameters and calibration data are in Table 1. These IBBS under-surveyed MSM aged ≥40 years (90% were aged <40), so the IBBS data were just used to calibrate the 18-39 age-group, with the same risk behaviours assumed for older MSM.

Age-related mortality data comes from UN databases(19) and we use existing population size estimates for MSM in Ukraine (~181,000(20)).

The model is seeded so that the age distribution of MSM in 1990 matches Ukraine’s male population, with MSM then entering and aging through the model. Although the HIV epidemic probably started later in Ukraine than Western Europe(21), there is considerable uncertainty around the initial epidemic, so we varied the number of seeded HIV-positive MSM in 1990 (0-2.8% prevalence).

In our previous analyses of the MSM IBBS data, being an NGO client was associated with(10):

- Increased condom usage during their last anal intercourse (Odds ratio [OR] 1.32; 95% confidence interval: 1.21-1.43),
- Increased HIV testing in last year (OR 7.01; 6.45-7.62),
- Increased coverage of ART (OR 2.20; 1.51-3.30).

We therefore assumed the rate of HIV-diagnosis and ART initiation are higher among NGO clients than non-NGO clients, and that they have higher condom use. This assumption is supported by NGO activities for MSM in Ukraine being focussed on providing HIV testing and counselling services, condom distribution, and linking HIV-positive people to ART services. NGOs also offer post-diagnosis case-management to MSM that test positive for HIV, incorporating counselling and linkage to ART. A detailed description of the assumptions made around the effects of NGOs is in the supplement (page 21).

NGOs targeting MSM initiated in Ukraine in 2003. Numbers from Alliance for Public Health (APH) indicate that there were 22,824 MSM registered with NGOs in 2013, 50,615 in 2018 (~28% of MSM), and stable thereafter, except for a COVID-19-related dip in 2020 (44,513 MSM). These numbers of MSM registered as NGO clients include those that receive at least the minimum package of services (including condom and information materials and/or consultation). They do not include other MSM that would have benefitted from NGO services such as condom distribution. The model was fit to these trends by assuming different rates of joining NGOs over time, with the rate increasing between 2003-2012 and 2013-2019 and then being constant thereafter, except in 2020 where a lower rate is assumed. Overall levels of condom use are assumed to have increased over 1990-2007(22), and then remain stable thereafter (as suggested by IBBS data), with higher condom use among NGO clients. ART became available in 2003, and available to all persons with HIV (PWH) in 2016(23), with rates of initiation fitted to increasing ART coverage levels among MSM (age 18-39) in 2011 and 2018 (IBBS data), differing by NGO status and assumed stable thereafter. HIV diagnosis data is available from 1990(24) with rates of diagnosis being fitted to the proportion of HIV-positive MSM (aged 18-39) that are HIV diagnosed in 2011 and 2018, which differs by NGO status.

We calibrated the model using an Approximate Bayesian computation sequential Monte Carlo (ABC SMC) scheme(25) to summary statistics (Table 1) among MSM aged 18-39 on the: proportion of MSM in contact with NGOs and the OR of being in contact with NGOs if HIV-positive versus HIV-negative; HIV prevalence in each combination of low- and high-risk NGO and non-NGO groups; and proportion of HIV-positive NGO and non-NGO clients that are diagnosed with HIV or on ART. The ABC SMC begins with 500 parameter sets sampled from prior distributions (Supplementary tables 1 and 2), which are successively perturbed to improve their goodness of fit, with this ceasing when there is no improvement between successive iterations. This produced 500 baseline model fits, which were used for all model analyses to produce a median estimate and 95% credibility intervals around that estimate, defined as the 2.5^th^-97.5^th^ percentile range.

The baseline model fits were cross-validated against overall HIV incidence (2013-2018(3, 16, 26)) and prevalence (2011-2018) estimates among MSM in Ukraine from the IBBS.

### Modelled impact analyses

We modelled the impact of existing coverage levels of NGOs (status quo) over 2016-2020, and if they were continued over 2021-2030, both compared to counterfactual scenarios where there was no effect of NGOs on condom use, HIV testing and initiating ART over these specific time periods, equivalent to NGOs ceasing activities and their effects stopping over these time periods. Although the recent invasion by Russia will have changed the status quo situation, it is too early to understand to what degree, with the counterfactual situation now also being relevant to show what could occur if services are disrupted compared to them being maintained. For the 2021-2030 scenario, we also varied the counterfactual to estimate the impact of each beneficial effect of the NGOs. We also estimated the impact over 2021-2030 of increasing the coverage of NGOs from 28% to 60% of MSM by 2025. Impact was estimated in terms of the number and percentage of HIV infections averted, percentage reduction in HIV incidence and HIV prevalence, and number and percentage of HIV deaths averted.

To investigate our assumptions about the effectiveness of NGOs, we also performed a sensitivity analysis in where we assumed not all the differences in condom use, HIV testing and ART coverage between NGO and non-NGO clients were due to NGO activities. For these, the counterfactual scenario incorporated different percentages of the difference in condom use, HIV testing, and linkage to HIV care between NGO and non-NGO clients. The status quo scenario was then compared to these scenarios. This can be interpreted as different percentages of the effects we are currently prescribing to NGOs being due to other causes.

### Cost-effectiveness analysis

Unit cost estimates for ART and NGO services for MSM (HIV counselling and testing, condom distribution, post-diagnosis HIV case management) came from published reports(27–29) and unpublished APH operational budget data for Ukraine (collected and provided by APH co-authors). Further details on the cost and health utility assumptions are given in the supplementary materials.

Due to the ongoing nature of NGO activities, we estimated the cost-effectiveness of current NGO activities by comparing the status quo scenario over 2016-2030 with a counterfactual scenario where NGO activities are stopped for 5 years (costs and effects of NGOs are removed) from 2016 and then restarted in 2021 until 2030. This allows us to capture some of the longer-term benefits of the NGO activities that occurred over 2016-2020(30, 31). This was done across all 500 baseline model fits (probabilistic sensitivity analysis or PSA) to produce a joint distribution for the incremental costs and DALYs between the intervention and counterfactual scenario, with all future costs and outcomes discounted at 3% per year. The mean incremental cost-effectiveness ratio (ICER) was calculated across the PSA outputs in terms of incremental cost per DALY averted. This was compared against a willingness-to-pay threshold of 50% of Ukraine’s per capita GDP in 2018 (US$3,096*0.5= US$1,548), the lowest estimated willingness-to-pay threshold for Ukraine based on health opportunity costs(33). Cost-effectiveness acceptability curves were plotted to determine the proportion of simulations that are cost-effective as a function of these willingness-to-pay thresholds. Sensitivity analyses included using different counterfactual scenarios incorporating different percentages of the effects of NGOs and are described further in the supplementary materials.

## Results

### Status quo model projections

**Figure 1:**
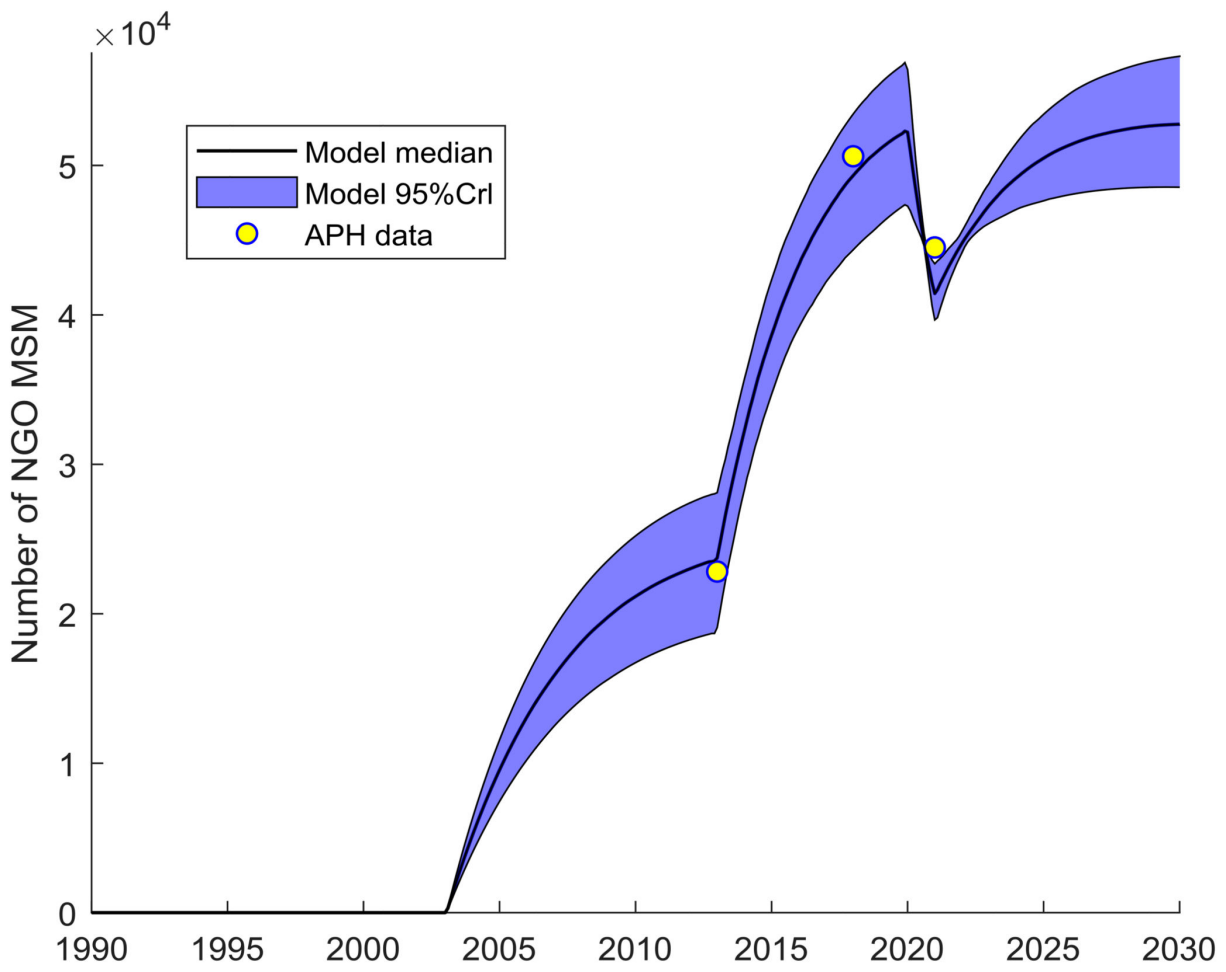
Data and model projections of the number of MSM that are NGO clients over time (status quo projections) MSM: men who have sex with men. NGO: Non-governmental organisation. 95%CrI: 95% credibility interval. APH: Alliance for Public Health, Ukraine.

**Figure 2:**
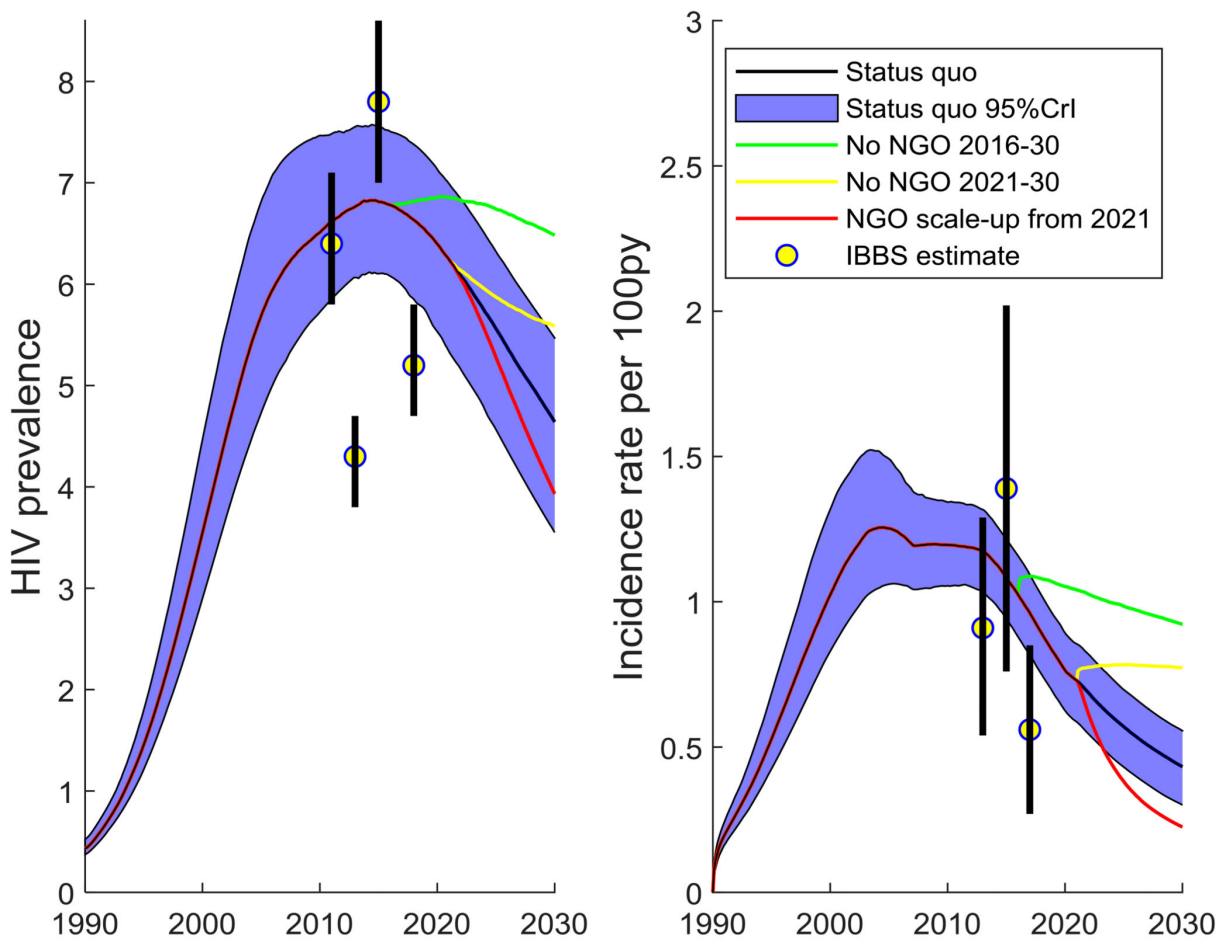
Overall HIV prevalence and incidence model projections for men who have sex with men in Ukraine from 1990-2030 for various intervention scenarios. Black line gives median model projections for the status quo scenario and blue shading gives the 95% credibility intervals. Overall HIV prevalence and incidence data are shown for comparison but were not used for fitting. MSM: men who have sex with men. NGO: Non-governmental organisation. 95%CrI: 95% credibility interval. IBBS: Integrated Bio-Behavioural Survey.

Figure 1 shows the modelled scale-up in NGO activities over time, fitted to APH data, with peak coverage reached around 2018, dropping in 2020 due to COVID-19. Supplementary figures 2 and 3 show that the model generally fitted the IBBS HIV prevalence and ART coverage data well. In addition, the model also agrees well with HIV incidence data not used in the model calibration, as shown in Figure 2.

With existing levels of NGO activity (supplementary table 3), where 28% (95%CrI: 26%-29%) of MSM are NGO clients in 2021, the model projects HIV incidence decreased from 1.23 (95%CrI: 1.02-1.51) per 100 person-years in 2003 when NGOs were introduced, to 0.73 (95%CI: 0.59-0.85) by 2021 and 0.43 (95%CrI: 0.30-0.56) by 2030 - a 41% reduction from 2021. HIV prevalence increased to 6.2% (95%CrI: 5.3%-7.0%) by 2021 from 4.9% (95%CrI: 4.1%-6.0%) in 2003, but will decrease to 4.6% (95%CrI: 3.6%-5.5%) by 2030; a 26% reduction. The model projects there were 6,253 (95%CrI: 5,587-6,954) HIV deaths and 6,151 (95%CrI: 5,133-7,008) HIV infections among MSM over 2016-2020 and 8,086 (95%CrI: 6,847-9,214) HIV deaths and 6,958 (95%CrI: 5,270-8,448) HIV infections over 2021-2030. The annual number of HIV-related deaths was estimated at 1,091 (95%CrI: 876-1,419) in 2003, and is projected to fall from 1,125 (95%CrI: 992-1255) in 2021 to 731 (95%CrI: 589-861) in 2030.

### Impact of historical NGO activities

**Figure 3:**
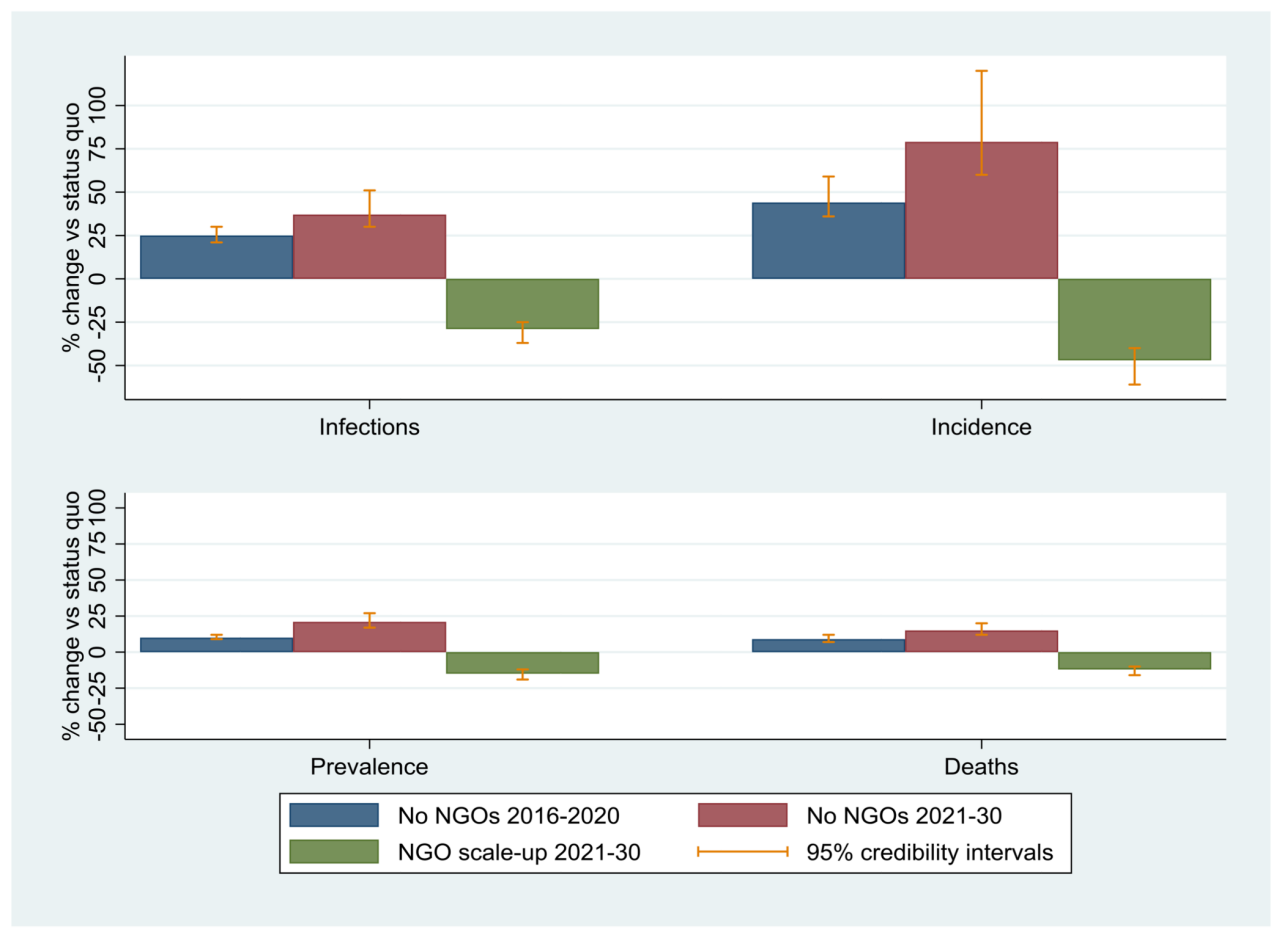
Percentage change in new HIV infections, HIV incidence, HIV prevalence, and HIV-related deaths among men who have sex with men for various intervention scenarios (compared to the status quo) over specified time periods. Whiskers denote the model 95% credibility intervals. NGO: Non-governmental organisation.

If there had been no NGO activity over 2016-2020 then HIV incidence would have been 44% (95%CrI: 36%-59%) higher in 2021, at 1.05 (95%CrI: 0.84-1.27) per 100 person-years (figure 2). There would have been 25% (95%CrI: 21%-30%) more HIV infections and 9% (95%CrI: 7%-12%) more HIV deaths over 2016-2020 (figure 3). Without NGO activities, the ART coverage among HIV-positive MSM would have been 25% (95%CI: 15%-31%) in 2021, instead of 42% (95%CI: 37%-47%) with status quo levels of NGO activities (supplementary figure 4).

When we consider the effect of removing specific beneficial effects of NGOs over 2016-2020, we find the number of HIV infections increases by 3% (95%CrI: 2%-6%) compared with the status quo scenario if we remove the increase in HIV diagnosis; 9% (95%CrI: 6%-14%) if we remove the increase in ART initiation; and 12% (95%CrI: 11%-14%) if we remove the increase in condom usage.

Lastly, supplementary table 4 shows that even if the counterfactual scenario incorporates half of the effects of NGOs over 2016-20, then having no NGO activities over this period would still have increased HIV incidence, HIV infections and deaths by 17% (15%-21%), 10% (9%-12%) and 4% (3%-4%), respectively.

### Impact of future NGO activities

If NGO activities were to cease over 2021-2030, then the number of incident HIV infections over this period would increase by 37% (95%CrI: 30%-51%) compared to the status quo scenario, HIV incidence in 2030 would be 79% (95%CrI: 60%-120%) higher, and 15% (95%CrI: 12%-20%) more deaths would occur (figure 3). Importantly, the increase in HIV incidence would occur quickly (Figure 3) highlighting that disruptions in NGO activities could quickly reverse decreasing trends in HIV incidence.

Alternatively, if NGO activities were scaled-up from 2021 such that double (~101,230) the number of MSM were clients of NGOs in 2025 than the status quo scenario (~50,615), then incidence decreases by half (47% lower; 95%CrI: 40%-61%) of what it would be in 2030 with the status quo scenario, to 0.23 (95%CrI: 0.14-0.30) per 100 person-years. This is 69% lower than it is currently estimated to be in 2020. HIV prevalence also decreases further to 3.9% (95%CrI: 3.0%-4.6%) in 2030, and there would be 29% (95%CrI: 25%-37%) fewer HIV infections and 12% (95%CrI: 10%-16%) fewer HIV deaths over 2021-2030 than for the status quo scenario.

### Cost-effectiveness of historical NGO activities

Compared to the counterfactual scenario with no effects of NGOs over 2016-2020 (but with NGO activities continuing over 2021-2030), the status quo scenario with NGOs in place over 2016-2030 is estimated to avert 3,025 (95%CrI: 2,343-4,092) HIV infections, 1,895 (95%CrI: 1,492-2,601) HIV deaths and 14,918 DALYs over 2016-2030. The status quo scenario costs US$35,600,695 (US$14,255,863 on ART and US$21,344,832 on NGOs), whilst the counterfactual scenario costs US$26,627,922 (US$12,661,237 on ART and US$13,986,685 on NGOs). The status quo scenario therefore incurs additional costs of US$8,952,733 over 2016-2030, resulting in a mean ICER of US$600.15 per DALY averted (Table 2 and supplementary table 6). The mean ICER is equivalent to 0.19 times the GDP per capita for Ukraine (US$3,096) with 100% of simulations in the PSA being cost-effective compared to the 0.5xGDP threshold, indicating this intervention is highly cost-effective at this willingness-to-pay threshold (Supplementary figure 5). The mean cost per death averted was US$4724.23 and the mean cost per HIV infection averted was US$2959.99.

The various one-way sensitivity analyses showed the cost-effectiveness results were robust, with the greatest mean ICER per DALY averted occurring when we assume higher costs for ART (US$972-$1,082 instead of the original assumptions of US$280.76-$312.53): US$863.95/DALY averted, which is still lower than the 0.5xGDP threshold, and so highly cost-effective. Conversely, the intervention becomes even more cost-effective (US$114/DALY averted) when a longer time horizon to 2050 is assumed. In the additional sensitivity analysis where different percentages of the effects prescribed to NGOs are assumed to be due to other causes (table 2), NGOs are still cost-effective at the 0.5xGDP threshold if 50% of the benefits of NGOs are assumed to be due to other reasons (US$1277.77).

## Discussions

Our modelling analyses suggest that without current NGO activities among MSM in Ukraine (over 2016-2020), the HIV incidence in 2021 would have been 44% higher and 25% more HIV infections would have occurred from 2016-2020. Going forward to 2030, continuing current NGO activities will reduce incidence by 40% and prevalence by 25% compared to what they are in 2021, whereas they would be 79% and 21% higher by 2030, respectively, if NGO activities cease. Importantly, most of the increase in HIV incidence occurs in the first year highlighting the importance of maintaining NGO activities despite the war with Russia because the public health implications of not doing so are stark. Doubling coverage of NGO activities from 2021 will further halve incidence by 2030. Projections suggest much of the ongoing benefit of NGO activities is through improving condom distribution and linkage to ART, with current NGO activities estimated to have increased the coverage of ART among HIV-positive MSM from 25% to 42% in 2021. Lastly, our analyses suggest that current NGO activities are highly cost-effective, costing US$600.15 per DALY averted, with all model projections and sensitivity analyses being lower than available willingness-to-pay thresholds for Ukraine.

### Comparison with literature

To our knowledge, this is the first study to examine the impact and cost-effectiveness of MSM-targeted NGOs in Ukraine. Wirtz et al previously modelled the impact of HIV prevention and treatment interventions among MSM in various low- and middle-income countries (LMIC), including Ukraine, and found that little impact on HIV incidence would be achieved unless ART was expanded(34). Unfortunately, little data for these analyses came from Ukraine and they did not calculate the cost-effectiveness of the interventions.

There has been limited research on Ukraine’s HIV epidemic among MSM. Our previous study used in this analysis found that NGOs were associated with beneficial outcomes among MSM(10), agreeing with other research(35). An MSM modelling study in Poland found that immediate initiation of HIV treatment was cost-saving from the public perspective(36). This was also a benefit of NGOs in Ukraine, enabling quicker diagnosis and linkage to HIV treatment. A review of spending on HIV in Eastern Europe and Asia found that programmes targeting MSM were moderately cost-effective, although there were few studies(37). Further afield, a study in Southern India showed that a large-scale intervention programme for HIV among MSM increased condom use, averted many HIV infections and was cost-effective(38). Other studies outside of Eastern Europe have examined the impact and cost-effectiveness of various non-PrEP interventions aimed at reducing HIV transmission among MSM, particularly in high-income countries(39–48), China(49–54), and other LMICs(55, 56). These studies generally show that targeted interventions for MSM can have large impact and be cost-effective.

The ICER per DALY averted for our study compares favourably with ICERs for other health interventions in Ukraine, particularly when considering this study’s ICER over a longer time horizon to 2050 (US$114). Other interventions in Ukraine with published ICERs include treatments for pneumonia (ICERs: US$910-1,317 per DALY averted)(57) scaling up opioid agonist treatment among PWID and expanding ART among the general population (ICERs: US$530-2,240 per DALY averted)(58, 59) or antenatal testing with a dual rapid test for HIV and syphilis (varying from cost-saving to $205 per DALY averted)(60).

### Strengths and limitations

The strength of our modelling includes using four rounds of national IBBS data to parameterise and calibrate the model within a Bayesian framework. Another strength is its novelty, with there being few evaluations of MSM-targeted interventions for Eastern Europe(61). Limitations include uncertainty around the MSM population size in Ukraine due to stigmatized around this population(4). Although we used the current best estimate for this parameter, there were limitations to the methods employed(20). There was uncertainty in other model parameters, which was incorporated into the model calibration, and the results were robust despite this. There was limited information on older MSM (aged ≥40) because they were not sampled well in the IBBS surveys.

The IBBS surveys are also limited because they are observational, and, so, could only be used to look at associations rather than causality in the epidemiological analyses that fed into this modelling. It is possible that some of our modelled intervention effects may result from more health-conscious people attending NGOs or from risk-behaviours differing between HIV-negative and HIV-positive MSM (HIV-positive MSM attended NGOs more than HIV-negative MSM), resulting in our projections possibly being overestimates. To counter this, we showed using stratified analyses that the beneficial associations of being an NGO client remained for both HIV-negative and HIV-positive MSM (supplementary page 21). Also, model sensitivity analyses found that NGOs still had considerable impact and were cost-effective even when we assumed some of the beneficial effects attributed to NGOs were due to other reasons. Conversely, we may be underestimating the impact of NGOs because our modelling only considers the effect of NGOs on their clients, rather than anyone reached by outreach services. Additionally, we did not incorporate the onward benefits of MSM interventions to their female partners. The perspective of this analysis is in terms of the payer (the Ukrainian government), however, the scope is only for ART, OST, and NGO costs, and do not include costs regarding health promotion or other healthcare costs related to PLHIV over time, as we did not have data to estimate them. In the 2018 IBBS report, viral suppression among HIV-positive MSM in Ukraine was 76% using a threshold of 1000 copies/mL(3), however, testing was performed upon a selected subgroup that self-reported being on ART, so we did not incorporate this into our analysis. Lastly, we incorporated the effect of COVID-19 on NGO interventions in 2020, suggesting a 10% decrease in MSM contacts.

### Conclusion

Our analyses suggest that NGOs targeting MSM in Ukraine are highly cost-effective and have been preventing considerable HIV infections and deaths. This beneficial impact has been achieved through condom distribution, while quickly diagnosing HIV-positive MSM and linking them to ART(9). Unfortunately, the Russian invasion is affecting the country’s response to the HIV epidemic(62). Although evidence suggests NGOs are still providing HIV services in many areas, HIV testing is reduced, and some regions are much worse affected. In the current situation, our findings are important for emphasising why these services should continue in Ukraine, while for other settings they are useful for guiding policymakers on how to optimally allocate their resources to achieve greatest health benefits. These analyses were undertaken at a time when Ukraine was undergoing a decrease in monetary support from the Global Fund(11) with funding transitioning to the Ukraine government. Even before Russia’s 2022 invasion, additional stresses were being placed on Ukraine’s economy by the war with Russia from 2014(63, 64) and the COVID-19 pandemic(65). Our analyses show that MSM HIV programming should continue, because otherwise the HIV epidemic among MSM will increase considerably. As stated by others(27), it is crucial that policymakers in Ukraine and other low- and middle-income countries are aware of the large, preventive effect that NGOs can and are having on HIV-transmission.

## Supplementary Material

Supplemental Data File (.doc, .tif, pdf, etc.)

## Figures and Tables

**Table 1:** Key model parameters and calibration data stratified by NGO status.

Variable	Non-NGO MSM		NGO MSM		All MSM	
Behavioural parameters	Low risk	High riskⱡ	Low risk	High riskⱡ	
Percentage of MSM using condom at last intercourse†	74%	62%	81%	68%	73%
Mean number of anal sex acts last month†	3.47	17.82	3.47	17.82	6.56
Mean number of anal sex partners last month†	2.03	3.75	2.03	3.75	2.40
**Epidemiological calibration parameters**					
HIV prevalence for MSM (2011)	5.5%	6.2%	6.9%	10.1%	6.4%†
HIV prevalence for MSM (2013)	3.2%	6.5%	6.2%	10.8%	4.3%†
HIV prevalence for MSM (2015)	5.2%	11.7%	8.1%	10.9%	7.8%†
HIV prevalence for MSM (2018)	4.0%	3.5%	7.2%	13.0%	5.2%†
**Intervention-related calibration parameters**					
Percentage of all HIV-positive MSM diagnosed 2011	17%	17%	34%	34%	23%†
Percentage of all HIV-positive MSM diagnosed 2018	33%	33%	58%	58%	45%†
Percentage of all HIV-positive MSM that are on ART 2011	3%	3%	28%	28%	7%†
Percentage of all HIV-positive MSM that are on ART 2018	15%	15%	51%	51%	39%†
Number of MSM in contact with NGOs in 2013	N/A	N/A	N/A	N/A	22824
Number of MSM in contact with NGOs in 2018	N/A	N/A	N/A	N/A	50615
Odds ratio of joining an NGO if HIV-positive vs HIV-negative	N/A	N/A	N/A	N/A	1.61

See Supplementary tables 1 and 2 for further information, 95% confidence intervals and data sources.

† Values for all MSM not used for model fitting.

ⱡ Defined as 10 or more sexual acts in a month

MSM: men who have sex with men. NGO: Non-governmental organisation.

**Table 2:** Sensitivity analyses on the incremental cost-effectiveness ratio over 2016-2030 (unless changed in sensitivity analysis) comparing the status quo with the scenario where there are no NGOs over 2016-2020, but they are resumed thereafter.

Scenario	Mean incremental cost-effectiveness ratio (US$)
Main comparison (status quo versus no NGOs 2016-2020)	$600.15
5% discount rate (instead of 3%)	$599.07
No discounting (instead of 3%)	$601.29
Time horizon to 2040 (instead of 2030)	$210.16
Time horizon to 2050 (instead of 2030)	$113.88
Alternative Deloitte costs for annual costs for HIV-negative and HIV-positive MSM in contact with NGOs (instead of APH data)†	$487.59
Alternative Deloitte costs for ART (instead of APH data)‡	$863.95
**Assuming different comparators to the status quo scenario:**	
Status quo versus 10% NGO effects 2016-2020*	$676.04
Status quo versus 20% NGO effects 2016-2020*	$770.55
Status quo versus 30% NGO effects 2016-2020*	$891.67
Status quo versus 40% NGO effects 2016-2020*	$1052.73
Status quo versus 50% NGO effects 2016-2020*	$1277.77
Status quo versus 60% NGO effects 2016-2020*	$1615.56
Status quo versus 70% NGO effects 2016-2020*	$2177.85
Status quo versus 80% NGO effects 2016-2020*	$3309.11
Status quo versus 90% NGO effects 2016-2020*	$6701.25

† Range: HIV-negative NGO clients $8.69-$21.26 and HIV-positive NGO clients $0.95-$6.36 instead of $17.00-$24.45 for all NGO clients.

‡ Range: $972-$1082 instead of $280.76-$312.53APH: Alliance for Public Health. ART: Antiretroviral therapy. NGO: Non-governmental organisations.

* Sensitivity analysis where we assumed not all the differences in condom use, HIV testing and ART coverage between NGO and non-NGO clients were due to NGO activities. For these, the counterfactual scenario incorporated different percentages of the difference in condom use, HIV testing, and linkage to HIV care between NGO and non-NGO clients. The status quo scenario was then compared to these scenarios. This can be interpreted as different percentages of the effects we are currently prescribing to NGOs being due to other causes.

## References

[1] UNAIDS (2016). Global AIDS Update 2016.

[2] ECDC (2018). HIV/AIDS surveillance in Europe.

[3] Alliance for Public Health (2019). Behavior monitoring of men having sex with men as a component of second generation surveillance.

[4] UNAIDS (2009). Hidden HIV epidemic amongst MSM in Eastern Europe and Central Asia.

[5] Holt E (2018). The Alliance for Public Health, Ukraine. Lancet Hiv.

[6] Foss AM, Watts CH, Vickerman P, Heise L (2004). Condoms and prevention of HIV. BMJ.

[7] Johnson WD, O’Leary A, Flores SA (2018). Per-partner condom effectiveness against HIV for men who have sex with men. Aids.

[8] Cohen MS, Chen YQ, McCauley M, Gamble T, Hosseinipour MC, Kumarasamy N (2016). Antiretroviral Therapy for the Prevention of HIV-1 Transmission. New Engl J Med.

[9] Rodger AJ, Cambiano V, Bruun T, Vernazza P, Collins S, Degen O (2019). Risk of HIV transmission through condomless sex in serodifferent gay couples with the HIV-positive partner taking suppressive antiretroviral therapy (PARTNER): final results of a multicentre, prospective, observational study. Lancet.

[10] Trickey A, Stone J, Semchuk N, Saliuk T, Sazonova I, Varetska O (2020). Is contact between men who have sex with men and non-governmental organisations providing harm reduction associated with improved HIV outcomes?. Hiv Med.

[11] PEPFAR (2018). Ukraine Country Operational Plan (COP) 2018-Strategic Direction Summary.

[12] AHF Ukraine Ukraine 2020.

[13] UNAIDS Country profile: Ukraine 2020.

[14] Hollingsworth TD, Anderson RM, Fraser C (2008). HIV-1 transmission, by stage of infection. J Infect Dis.

[15] Klepikov A (2019). Health. AfP. PrEP Experience in Ukraine.

[16] Alliance for Public Health (2017). Analytical report: Monitoring of behaviour and HIV prevalence among men having sex with men (national part).

[17] International HIV/AIDS Alliance in Ukraine (2012). Analytical report: Behaviour monitoring and HIV-prevalence among men who have sex with men as a component of second generation surveillance.

[18] International HIV/AIDS Alliance in Ukraine (2014). Summary of the analytical report: Monitoring the behaviour and HIV-infection prevalence among men who have sex with men as a component of HIV second generation surveillance.

[19] United Nations Department of Economic and Social Affairs (2020). Mortality estimates.

[20] Alliance for Public Health (2018). Estimation of the Size of Populations Most-at-Risk for HIV Infection in Ukraine.

[21] Pope C, White RT, Malow R (2009). HIV/AIDS : global frontiers in prevention/intervention.

[22] US Centers for Disease Control (2003). Chapter 14: Reproductive, Maternal and Child Health in Eastern Europe and Eurasia: A Comparative Report.

[23] World Health Organization (2015). Guideline on when to start antiretroviral therapy and on preexposure prophylaxis for HIV.

[24] Twigg J (2006). HIV/AIDS in Russia and Eurasia.

[25] Sunnaker M, Busetto AG, Numminen E, Corander J, Foll M, Dessimoz C (2013). Approximate Bayesian computation. PLoS Comput Biol.

[26] Alliance for Public Health (2014). Summary of the analtical report: Monitoring the behaviour and HIV-infection prevalence among men who have sex with men as a component of HIV second generation surveillance.

[27] Latypov A, Dierst-Davies R, Sereda Y, Kerr CC, Duda M, Deloitte (2018). HIV investment case study for Ukraine: Evaluation of program costs, service quality, and resource allocation for HIV expenditure in 2015.

[28] Optima (2020). Resource optimization to maximize the HIV response in Eastern Europe and Central Asia.

[29] The USAID HIV Reform in Action Project (2018). HIV Investment case study for Ukraine: Evaluation of program costs, service quality, and resource allocation for HIV expenditure in 2015.

[30] Sweeney S, Ward Z, Platt L, Guinness L, Hickman M, Hope V (2019). Evaluating the costeffectiveness of existing needle and syringe programmes in preventing hepatitis C transmission in people who inject drugs. Addiction.

[31] Stone J, Walker JG, Bivegete S, Trickey A, Semchuk N, Sazonova Y Evaluating the impact and cost-effectiveness of existing HIV prevention interventions among people who inject drugs in Ukraine. In preparation.

[32] Bertram MY, Lauer JA, De Joncheere K, Edejer T, Hutubessy R, Kieny MP (2016). Costeffectiveness thresholds: pros and cons. B World Health Organ.

[33] Ochalek J, Lomas J, Claxton K (2018). Estimating health opportunity costs in low-income and middle-income countries: a novel approach and evidence from cross-country data. BMJ Glob Health.

[34] Wirtz AL, Walker DG, Bollinger L, Sifakis F, Baral S, Johns B (2013). Modelling the impact of HIV prevention and treatment for men who have sex with men on HIV epidemic trajectories in low-and middle-income countries. Int J Std Aids.

[35] Iakunchykova O, Burlaka V, King EJ (2018). Correlates of Serosorting and Knowledge of Sexual Partner’s HIV Status Among Men Who have Sex with Men in Ukraine. Aids Behav.

[36] Kowalska JD, Wojcik G, Rutkowski J, Ankiersztejn-Bartczak M, Siewaszewicz E (2017). Modelling the cost-effectiveness of HIV care shows a clear benefit when transmission risk is considered in the calculations-A message for Central and Eastern Europe. Plos One.

[37] Craig AP, Thein HH, Zhang L, Gray RT, Henderson K, Wilson D (2014). Spending of HIV resources in Asia and Eastern Europe: systematic review reveals the need to shift funding allocations towards priority populations. Journal of the International Aids Society.

[38] Ramanathan S, Deshpande S, Gautam A, Pardeshi DB, Ramakrishnan L, Goswami P (2014). Increase in condom use and decline in prevalence of sexually transmitted infections among high-risk men who have sex with men and transgender persons in Maharashtra, India: Avahan, the India AIDS Initiative. BMC Public Health.

[39] Zulliger R, Maulsby C, Solomon L, Baytop C, Orr A, Nasrullah M (2017). Cost-utility of HIV Testing Programs Among Men Who Have Sex with Men in the United States. Aids Behav.

[40] De P, Downing MJ, Hirshfield S (2018). Cost Analysis of Implementing a Video-Based Ehealth Intervention for Hiv-Positive Gay, Bisexual, and Other Men Who Have Sex with Men. Aids Educ Prev.

[41] Bom RJM, van der Linden K, Matser A, Poulin N, van der Loeff MFS, Bakker BHW (2019). The effects of free condom distribution on HIV and other sexually transmitted infections in men who have sex with men. Bmc Infect Dis.

[42] Irvine MA, Konrad BP, Michelow W, Balshaw R, Gilbert M, Coombs D (2018). A novel Bayesian approach to predicting reductions in HIV incidence following increased testing interventions among gay, bisexual and other men who have sex with men in Vancouver, Canada. J R Soc Interface.

[43] Palk L, Gerstoft J, Obel N, Blower S (2018). A modeling study of the Danish HIV epidemic in men who have sex with men: travel, pre-exposure prophylaxis and elimination. Sci Rep-Uk.

[44] Reitsema M, Heijne J, Visser M, van Sighem A, van der Loeff MS, op de Coul ELM (2020). Impact of frequent testing on the transmission of HIV and N. gonorrhoeaeamong men who have sex with men: a mathematical modelling study. Sex Transm Infect.

[45] Reitsema M, Steffers L, Visser M, Heijne J, van Hoek AJ, van der Loeff MS (2019). Costeffectiveness of increased HIV testing among MSM in The Netherlands. Aids.

[46] van Sighem A, Vidondo B, Glass TR, Bucher HC, Vernazza P, Gebhardt M (2012). Resurgence of HIV Infection among Men Who Have Sex with Men in Switzerland: Mathematical Modelling Study. Plos One.

[47] Reback CJ, Fletcher JB, Leibowitz AA (2019). Cost effectiveness of text messages to reduce methamphetamine use and HIV sexual risk behaviors among men who have sex with men. J Subst Abuse Treat.

[48] Mitchell KM, Hoots B, Dimitrov D, German D, Flynn C, Farley JE (2019). Improvements in the HIV care continuum needed to meaningfully reduce HIV incidence among men who have sex with men in Baltimore, US: a modelling study for HPTN 078. Journal of the International Aids Society.

[49] Wang KR, Peng LP, Gu J, Hao C, Zou HC, Hao YT (2018). Impact of the 90-90-90 goal and preexposure prophylaxis on HIV transmission and elimination in men who have sex with men in China: A mathematical modeling study. Zhonghua Liu Xing Bing Xue Za Zhi.

[50] Li JH, Peng LP, Gilmour S, Gu J, Ruan YH, Zou HC (2018). A mathematical model of biomedical interventions for HIV prevention among men who have sex with men in China. Bmc Infect Dis.

[51] Hu QH, Meyers K, Xu JJ, Chu ZX, Zhang J, Ding HB (2019). Efficacy and cost-effectiveness of early antiretroviral therapy and partners’ pre-exposure prophylaxis among men who have sex with men in Shenyang, China: a prospective cohort and costing study. Bmc Infect Dis.

[52] Sun XD, Xiao YN, Peng ZH, Wang N (2013). Modelling HIV/AIDS Epidemic among Men Who Have Sex with Men in China. Biomed Res Int.

[53] Zhuang X, Peng P, Sun HM, Chu MJ, Jiang SY, Jiang LY (2018). Scaling Up Human Immunodeficiency Virus Screening and Antiretroviral Therapy Among Men Who Have Sex With Men to Achieve the 90-90-90 Targets in China. Sexually Transmitted Diseases.

[54] Huang SZ, Dai WC, Li XF, Jiang XH, Tang WM, Zhou Y (2020). Cost-effectiveness of HIV self-testing strategy in men who have sex with men. Zhonghua Liu Xing Bing Xue Za Zhi.

[55] Caro-Vega Y, del Rio C, Lima VD, Lopez-Cervantes M, Crabtree-Ramirez B, Bautista-Arredondo S (2015). Estimating the Impact of Earlier ART Initiation and Increased Testing Coverage on HIV Transmission among Men Who Have Sex with Men in Mexico using a Mathematical Model. Plos One.

[56] Ifekandu C, Suleiman A, Aniekwe O (2014). The cost-effectiveness in the use of HIV counselling and testing-mobile outreaches in reaching men who have sex with men (MSM) in northern Nigeria. Journal of the International Aids Society.

[57] Iakovlieva L, Bezditko N, Glumcher F, Dubrov F (2013). Cost-Effectiveness Analysis of Carbapenems in Treatment Nosocomian Pneumonia in Ukraine. Value Health.

[58] Alistar SS, Owens DK, Brandeau ML (2011). Effectiveness and Cost Effectiveness of Expanding Harm Reduction and Antiretroviral Therapy in a Mixed HIV Epidemic: A Modeling Analysis for Ukraine. Plos Med.

[59] Morozova O, Crawford FW, Cohen T, Paltiel AD, Altice FL (2020). Cost-effectiveness of expanding the capacity of opioid agonist treatment in Ukraine: dynamic modeling analysis. Addiction.

[60] Rodriguez PJ, Roberts DA, Meisner J, Sharma M, Owiredu MN, Gomez B (2021). Costeffectiveness of dual maternal HIV and syphilis testing strategies in high and low HIV prevalence countries: a modelling study. Lancet Glob Health.

[61] Croxford S, Tavoschi L, Sullivan AK, Combs L, Raben D, Delpech V (2020). HIV testing strategies outside of health care settings in the European Union (EU)/European Economic Area (EEA): a systematic review to inform European Centre for Disease Prevention and Control guidance. Hiv Med.

[62] Holt E (2022). Russia’s invasion of Ukraine threatens HIV response. Lancet Hiv.

[63] Clark D (2017). Ukraine’s economy has turned a corner. The Financial Times.

[64] Vasylyeva TI, Liulchuk M, Friedman SR, Sazonova I, Faria NR, Katzourakis A (2018). Molecular epidemiology reveals the role of war in the spread of HIV in Ukraine. P Natl Acad Sci USA.

[65] Organisation for Economic Co-operation and Development (2020). The COVID-19 crisis in Ukraine.

