# Supplementary material for: Impact and cost-effectiveness of non-governmental organizations on the HIV epidemic in Ukraine among men who have sex with men
Source: AIDS. Author manuscript; Available in PMC 2022 Nov 15. (PMC7613764; doi:10.1097/QAD.0000000000003347)
Supplement: Supplemental Data File (.doc, .tif, pdf, etc.) [file EMS151903-supplement-Supplemental_Data_File___doc___tif__pdf__etc__.docx]

**Impact and cost-effectiveness of non-governmental organizations on the HIV epidemic in Ukraine among men who have sex with men**

Adam TRICKEY, Josephine G WALKER, Sandra BIVEGETE, Nadiya SEMCHUK, Tetiana SALIUK, Olga VARETSKA, Jack STONE, Peter VICKERMAN

**Supplementary materials**

**Contents:**

Page 2 - Supplementary table 1: Prior distributions and sources for model parameters.

Page 5 - Supplementary table 2: Parameters that the models are fit to (with 95% confidence intervals) and further model parameters.

Page 7 - Force of infection and model equations

Page 16 - Supplementary figure 1: Model schematics of how MSM transition through (a) age, low/high-risk sexual behaviour, and NGO status groups; b) different stages of HIV progression.

Page 17 - Supplementary figure 2: Data and model projections of the number of MSM that are NGO clients (status quo projections)

Page 18 - Supplementary figure 3: A comparison of data with our modelled HIV prevalence projections stratified by low/high risk group and NGO status for MSM aged 18-39 years.

Page 19 - Supplementary figure 4: The proportion of HIV-positive MSM aged 18-39 that are on ART stratified by NGO client status.

Page 20 - Supplementary figure 5: The proportion of HIV-positive MSM that have been diagnosed with HIV, and the proportion of HIV-positive MSM that are on ART, for various scenarios from 1990-2030.

Page 21 - Investigating assumptions about NGO effectiveness

Page 22 - Supplementary table 3: Model results for selected scenarios over different time periods, compared to a scenario where NGOs are 50% less effective – median (95% credibility intervals).

Page 23 - Cost and health utility assumptions

Page 24 - Supplementary table 4: Unit costs of antiretroviral therapy (ART) and NGO services provided for MSM in Ukraine, in 2018 US dollars (US$)

Page 25 - Supplementary table 5: Mean incremental costs, DALYs and incremental cost-effectiveness ratio over 2016-2030 for the status quo compared to a counterfactual scenario where there are no NGOs over 2016-2020 but they resume thereafter.

Page 25 - Supplementary figure 6: Probabilistic sensitivity analysis of the cost-effectiveness acceptability curve of the status quo scenario vs no non-governmental organisations for 2016-2020.

Page 26 - References

**Supplementary table 1:** Prior distributions and posterior distributions and sources for model parameters.

| **Parameter** | **Prior** | **Posterior (range)** | **Source** |
| --- | --- | --- | --- |
| Number of anal sex acts for low risk MSM | Norm(3.47, 0.018) [Truncated to 95% CI] | 3.470-3.4997 | IBBS data (2011, 2013, 2015, 2018) analysis for this paper - (1) |
| Number of anal sex acts for high risk MSM | Norm(17.82, 0.169) [Truncated to 95% CI] | 17.4909-17.8461 | IBBS data (2011, 2013, 2015, 2018) analysis for this paper - (1) |
| Number of anal sex partners for low risk MSM | Norm(2.03, 0.016) [Truncated to 95% CI] | 2.0121-2.0299 | IBBS data (2011, 2013, 2015, 2018) analysis for this paper - (1) |
| Number of anal sex partners for high risk MSM | Norm(3.75, 0.061) [Truncated to 95% CI] | 3.7863-3.8514 | IBBS data (2011, 2013, 2015, 2018) analysis for this paper - (1) |
| Proportion of condom use for low-risk non-NGO MSM | Norm(0.74, 0.01) [Truncated to 95% CI] | 0.7418-0.7467 | IBBS data (2011, 2013, 2015, 2018) analysis for this paper - (1) |
| Proportion of condom use for high-risk non-NGO MSM | Norm(0.62, 0.01) [Truncated to 95% CI] | 0.6002-0.6083 | IBBS data (2011, 2013, 2015, 2018) analysis for this paper - (1) |
| Proportion of condom use for low-risk NGO MSM | Norm(0.81, 0.01) [Truncated to 95% CI] | 0.8053-0.8198 | IBBS data (2011, 2013, 2015, 2018) analysis for this paper - (1) |
| Proportion of condom use for high-risk NGO MSM | Norm(0.68, 0.01) [Truncated to 95% CI] | 0.6906-0.7067 | IBBS data (2011, 2013, 2015, 2018) analysis for this paper - (1) |
| Efficacy of condom use for each sex act | Triangular(0.69, 0.91, 1.00) | 0.8371-0.9229 | Johnson WD, O’Leary A, Flores SA. Per-partner condom effectiveness against HIV for men who have sex with men. AIDS. 2018;32(11):1499-1505. - (2) |
| Ratio of proportion of condom use in 1990 vs 2007 | Uniform(0.1, 1) | 0.5455-0.7885 | Uncertain data on condom use among MSM over time. Lower limit based on data for Romania for women aged 15-24 showing it rose from 4% in 1993 to 39% in 1999 – US Centers for Disease Control. Reproductive, Maternal and Child Health in Eastern Europe and Eurasia: A Comparative Report. Chapter 14. 2003. - (3) |
| Rate of stopping ART per year | Triangular(0.0425, 0.0432, 0.0439) | 0.0428-0.0432 | Analyses of Antiretroviral cohort collaboration (ART-CC) data on loss to follow-up among MSM (1996-2018) - (4) |
| Rate of starting ART 2003-2010 for non-NGO MSM per year | Uniform(0, 3) | 0.0002-0.1371 | No data available so using uninformative prior – estimated through model calibration |
| Rate of starting ART 2003-2010 for NGO MSM per year | Uniform(0, 3) | 0.8774-1.5743 | No data available so using uninformative prior – estimated through model calibration |
| Rate of starting ART 2011-2017 for non-NGO MSM per year | Uniform(0, 3) | 0.0276-0.4377 | No data available so using uninformative prior – estimated through model calibration |
| Rate of starting ART 2011-2017 for NGO MSM per year | Uniform(0, 3) | 1.2499-2.1697 | No data available so using uninformative prior – estimated through model calibration |
| Rate of HIV diagnosis 1990-2010 for non-NGO MSM per year | Uniform(0, 1) | 0.0266-0.0753 | No data available so using uninformative prior – estimated through model calibration |
| Rate of HIV diagnosis 1990-2010 for NGO MSM per year | Uniform(0, 1) | 0.0612-0.1925 | No data available so using uninformative prior – estimated through model calibration |
| Rate of HIV diagnosis 2011-2017 for non-NGO MSM per year | Uniform(0, 1) | 0.0769-0.2099 | No data available so using uninformative prior – estimated through model calibration |
| Rate of HIV diagnosis 2011-2017 for NGO MSM per year | Uniform(0, 1) | 0.2134-0.4690 | No data available so using uninformative prior – estimated through model calibration |
| Rate of joining NGOs if HIV- 2003-2012 per year | Uniform(0, 0.25) | 0.0234-0.0448 | No data available so using uninformative prior – estimated through model calibration |
| Rate of joining NGOs if HIV- post-2013 per year | Uniform(0, 0.25) | 0.0825-0.1286 | No data available so using uninformative prior – estimated through model calibration |
| Rate of joining NGOs if HIV+ 2003-2012 per year | Uniform(0, 0.25) | 0.0368-0.0764 | No data available so using uninformative prior – estimated through model calibration |
| Rate of joining NGOs if HIV+ post-2013 per year | Uniform(0, 0.25) | 0.1399-0.2018 | No data available so using uninformative prior – estimated through model calibration |
| Rate of leaving NGOs per year | Uniform(0.0877, 0.1756) | 0.1299-0.1682 | Alliance for Public Health data on number of clients per year - (1) |
| Proportion of MSM that start as low risk | Norm(0.778, 0.003) | 0.7827-0.7877 | IBBS data (2011, 2013, 2015, 2018) analysis for this paper- (1) |
| Rate of going from low risk to high risk, aged 18-39 per year* | Uniform(0, 0.25) | 0.1752-0.2499 | No data available so using prior with large uncertainty |
| Rate of going from low risk to high risk, aged 40+ per year* | Uniform(0, 0.25) | 0.0045-0.0758 | No data available so using prior with large uncertainty |
| Death rate for 18-39-year olds per year | Uniform(0.00211, 0.00484) | 0.0029-0.0036 | UN data - (5) |
| Death rate for ≥40-year olds per year | Uniform(0.02993, 0.0397) | 0.0337-0.0378 | UN data - (5) |
| Months from AIDS to death | LogNorm(10, 6.79, 12.7) | 8.7798-11.1947 | Morgan D, Mahe C, Mayanja B, Okongo JM, Lubega R, Whitworth JA. HIV-1 infection in rural Africa: is there a difference in median time to AIDS and survival compared with that in industrialized countries? AIDS. 2002;16(4):597-603. - (6) |
| Months from pre-AIDS to AIDS | Triangular(4.81, 9, 14) | 8.2690-12.8199 | Hollingsworth TD, Anderson RM, Fraser C. HIV-1 transmission, by stage of infection. J Infect Dis. 2008;198(5):687-93. - (7) |
| Months from acute to chronic HIV | Triangular(1.23, 2.9, 6.0) | 1.2346-2.7837 | Hollingsworth TD, Anderson RM, Fraser C. HIV-1 transmission, by stage of infection. J Infect Dis. 2008;198(5):687-93. - (7) |
| Years from infection to AIDS | Triangular(5.5, 9.4, 10.1) | 5.5528-6.5825 | Morgan D, Mahe C, Mayanja B, Okongo JM, Lubega R, Whitworth JA. HIV-1 infection in rural Africa: is there a difference in median time to AIDS and survival compared with that in industrialized countries? AIDS. 2002;16(4):597-603. - (6) |
| Relative rate of HIV progression if on ART | Uniform(0, 0.5) | 0.3751-0.4992 | Taking a combined range from:  Sucharitakul K, Boily M, Dimitrov D, Mitchell KM. Influence of model assumptions about HIV disease progression after initiating or stopping treatment on estimates of infections and deaths averted by scaling up antiretroviral therapy. PLoS One. 2018;13(3). - (8)  AND  Cori A, Ayles H, Beyers N, Schaap A, et al. HPTN 071 (PopART): A Cluster-Randomized Trial of the Population Impact of an HIV Combination Prevention Intervention Including Universal Testing and Treatment: Mathematical Model. PLoS One. 2014;9(1). - (9) |
| Transmissibility for acute stage | LogNorm(276, 131, 509) | 157.0436-258.3913 | Hollingsworth TD, Anderson RM, Fraser C. HIV-1 transmission, by stage of infection. J Infect Dis. 2008;198(5):687-93. - (7) |
| Transmissibility for pre-AIDS stage | LogNorm(76, 41.3, 128.0) | 41.4520-75.5016 | Hollingsworth TD, Anderson RM, Fraser C. HIV-1 transmission, by stage of infection. J Infect Dis. 2008;198(5):687-93. - (7) |
| Transmissibility for chronic stage | LogNorm(10.6, 7.61, 13.3) | 9.9522-12.1147 | Hollingsworth TD, Anderson RM, Fraser C. HIV-1 transmission, by stage of infection. J Infect Dis. 2008;198(5):687-93. - (7) |
| HIV transmission beta | Uniform(0, 0.05) | 0.0082-0.0148 | No data available so using uninformative prior – estimated through model calibration |
| Ratio of transmissibility for being on ART vs not | Uniform(0.01, 0.4) | 0.0268-0.1550 | Berenguer J, Parrondo J, Landovitz RJ. Mathematical modeling of HIV-1 transmission risk from condomless anal intercourse in HIV-infected MSM by the type of initial ART. 2019;14(7). - (10) |
| HIV-positive MSM in 1990 | Triangular(10, 500, 2000) | 544-980 | No data available so using prior with large uncertainty |

NGO: Non-governmental organisation. MSM: men who have sex with men. APH: Alliance for Public Health. CI: Confidence Interval. IBBS: Integrated Bio-Behavioural Survey. ART: Antiretroviral Therapy.

*Rates of going from high risk to low risk per year, aged 18-39 and aged 40+ are calculated by balancing the numbers of MSM going from low to high risk so that the proportion considered high risk is stable over time.

**Supplementary table 2:** Parameters that the models are fit to (with 95% confidence intervals†) and further model parameters.

| **Fitting parameter** | **Value fitted to (95% confidence intervals)** | **Source** |
| --- | --- | --- |
| Number of MSM in contact with NGOs 2013 | 22824 | APH data on total number of MSM registered with them |
| Number of MSM in contact with NGOs 2018 | 50615 | APH data on total number of MSM registered with them |
| Number of MSM in contact with NGOs 2020 | 44513 | APH data on total number of MSM registered with them |
| HIV prevalence low-risk non-NGO MSM aged 18-39 2011 | 5.5% (5%-6%) | IBBS data analysis for this paper |
| HIV prevalence low-risk non-NGO MSM aged 18-39 2013 | 3.2% (3%-4%) | IBBS data analysis for this paper |
| HIV prevalence low-risk non-NGO MSM aged 18-39 2015 | 5.2% (4%-6%) | IBBS data analysis for this paper |
| HIV prevalence low-risk non-NGO MSM aged 18-39 2018 | 4.0% (3%-5%) | IBBS data analysis for this paper |
| HIV prevalence high-risk non-NGO MSM aged 18-39 2011 | 6.2% (5%-7%) | IBBS data analysis for this paper |
| HIV prevalence high-risk non-NGO MSM aged 18-39 2013 | 6.5% (3%-11%) | IBBS data analysis for this paper |
| HIV prevalence high-risk non-NGO MSM aged 18-39 2015 | 11.7% (9%-14%) | IBBS data analysis for this paper |
| HIV prevalence high-risk non-NGO MSM aged 18-39 2018 | 3.5% (2%-5%) | IBBS data analysis for this paper |
| HIV prevalence low-risk NGO MSM aged 18-39 2011 | 6.9% (5%-9%) | IBBS data analysis for this paper |
| HIV prevalence low-risk NGO MSM aged 18-39 2013 | 6.2% (5%-7%) | IBBS data analysis for this paper |
| HIV prevalence low-risk NGO MSM aged 18-39 2015 | 8.1% (7%-10%) | IBBS data analysis for this paper |
| HIV prevalence low-risk NGO MSM aged 18-39 2018 | 7.2% (6%-9%) | IBBS data analysis for this paper |
| HIV prevalence high-risk NGO MSM aged 18-39 2011 | 10.1% (8%-13%) | IBBS data analysis for this paper |
| HIV prevalence high-risk NGO MSM aged 18-39 2013 | 10.8% (5%-19%) | IBBS data analysis for this paper |
| HIV prevalence high-risk NGO MSM aged 18-39 2015 | 10.9% (8%-14%) | IBBS data analysis for this paper |
| HIV prevalence high-risk NGO MSM aged 18-39 2018 | 13.0% (10%-16%) | IBBS data analysis for this paper |
| Proportion HIV-positive non-NGO MSM aged 18-39 diagnosed 2011 | 0.173 (0.13-0.23) | IBBS data analysis for this paper |
| Proportion HIV-positive non-NGO MSM aged 18-39 diagnosed 2018 | 0.329 (0.26-0.41) | IBBS data analysis for this paper |
| Proportion HIV-positive NGO MSM aged 18-39 diagnosed 2011 | 0.336 (0.25-0.43 | IBBS data analysis for this paper |
| Proportion HIV-positive NGO MSM aged 18-39 diagnosed 2018 | 0.580 (0.50-0.66) | IBBS data analysis for this paper |
| Proportion HIV-positive non-NGO MSM aged 18-39 on ART 2011 | 0.032 (0.014-0.061) | IBBS data analysis for this paper |
| Proportion HIV-positive non-NGO MSM aged 18-39 on ART 2018 | 0.152 (0.094-0.227) | IBBS data analysis for this paper |
| Proportion HIV-positive NGO MSM aged 18-39 on ART 2011 | 0.280 (0.212-0.356) | IBBS data analysis for this paper |
| Proportion HIV-positive NGO MSM aged 18-39 on ART 2018 | 0.513 (0.430-0.596) | IBBS data analysis for this paper |
| Odds ratio of joining NGOs for HIV-positive versus HIV-negative MSM | 1.61 (1.39-1.86) | IBBS data analysis for this paper |
| **Other parameter** | **Value used** | **Source** |
| MSM population size (used for 1990) | 181000 | APH. Estimation of the Size of Populations Most-at-Risk for HIV Infection in Ukraine - (11) |
| Proportion of population aged ≥18 that are ≥40 years old | 0.783 | UN data |
| Disability weight for MSM with acute HIV, chronic HIV, or on ART | 0.078 (Triangular: 0.052-0.111) | Salomon JA, Haagsma JA, Davis A, et al. Disability weights for the Global Burden of Disease 2013 study. 2015; 3 (11) - (12) |
| Disability weight for MSM with pre-AIDS HIV, not on ART | 0.274 (Triangular: 0.184-0.377) | Salomon JA, Haagsma JA, Davis A, et al. Disability weights for the Global Burden of Disease 2013 study. 2015; 3 (11) - (12) |
| Disability weight for MSM with AIDS, not on ART | 0.582 (Triangular: 0.406-0.743) | Salomon JA, Haagsma JA, Davis A, et al. Disability weights for the Global Burden of Disease 2013 study. 2015; 3 (11) - (12) |

†The 95% confidence intervals are not used for fitting.

NGO: Non-governmental organisation. MSM: men who have sex with men. APH: Alliance for Public Health. IBBS: Integrated Bio-Behavioural Survey. ART: Antiretroviral Therapy.

**Force of infection and model equations**

For each sexual partner that someone has, the force of infection (FOI) is estimated by firstly calculating the probability that they form a sexual partnership with someone from the low or high sexual risk group. This is then multiplied by the probability that an individual in that risk group is in each different infection class (multiplied by any associated HIV transmission cofactor for that stage of infection), which is then multiplied by the rate of being infected by that person in each time unit. This last rate is estimated by the rate of sexual acts that this partnership has, multiplied by the chance that each of these sex acts is not protected by a condom (using the average of the condom use reported by each side of the sexual partnership), further multiplied by the chance of HIV transmission in one unprotected sex act. Then, because this equation is for one sexual partner that the index individual has, it should be multiplied by the number of sexual partners they have per time unit.

MSM: Men who have sex with men

NGO: Non-governmental organisation

ART: Antiretroviral therapy

**HIV transmission equations**

The FOI for an HIV-negative MSM in risk group r and NGO group n:

$\vartheta_{rn}=\beta P_{r}\sum_{all i j} [\frac{m_{r}+m_{i}}{2}]\left[ \frac{{{Group}_{ij}P}_{i}}{\sum_{all lk} {{Group}_{lk}P}_{l}} \right]\left[ 1-G\frac{\left( {Con}_{rn}+{Con}_{ij} \right)}{2} \right]\left( \frac{A_{ij}E+C_{ij}+O_{ij}DU+T_{ij}U+F_{ij}D}{Z_{ij}} \right)$

$Z_{rn}$ is the number of MSM in risk group r and NGO group n that are sexually active, including susceptibles, infected, and those on ART, whilst excluding those with AIDS that are not on ART, where:

$Z_{rn}=A_{rn}+C_{rn}+T_{rn}+F_{rn}+S_{rn}$

$A_{rn}$= number of MSM acutely infected in risk group r and NGO group n

$C_{rn}$= number of MSM Chronic and not on ART with chronic HIV in risk group r and NGO group n

$T_{rn}$ = number of MSM with chronic HIV and on ART in risk group r and NGO group n

$O_{rn}$ = number of MSM pre-AIDS and on ART in risk group r and NGO group n

$F_{rn}$= number of MSM with pre-AIDS and not on ART

$S_{rn}$ = number susceptible to HIV infection but not on PrEP for MSM in risk group r and NGO status n

$\beta$ = transmission factor for HIV per sex act taken from systematic reviews as the average of insertive and receptive

$P_{r}$ = number of partners per month for risk group r (not dependent on NGO status n)

$m_{r}$ = number of sex acts on average per partner per month dependent on the individual’s risk group (acts/partners) (but not NGO status n)

${Group}_{rn}$ = number of MSM in each NGO/risk combination category

$G$ = efficacy of condom use for each sex act

${Con}_{rn}$ = average consistency of condom use that varies by risk group and NGO status

$E$ = transmissibility for acute stage vs “Chronic”

$D$ = transmissibility for pre-AIDS vs “Chronic”

$U$ = transmissibility for those on ART vs “Chronic”

**Full model equations**

Subscripts 1-8 indicate the age group, risk group, and NGO status: 1 for age 18-39, low-risk, non-NGO MSM, 2 for age ≥40, low-risk, non-NGO MSM, 3 for age 18-39, low-risk, NGO MSM, 4 for age ≥40, low-risk, NGO MSM, 5 for age 18-39, high-risk, non-NGO MSM, 6 for age ≥40, high-risk, non-NGO MSM, 7 for age 18-39, high-risk, NGO MSM, 8 for age ≥40, high-risk, NGO MSM.

Proportion low-risk at age 18 = $\rho_{1}$

Proportion high-risk at age 18 = $\rho_{2}$

Rate of joining NGOs = ω

Rate of leaving NGOs = τ

Rate of going from high-risk to low-risk aged 18-39 years = $\lambda_{1}$

Rate of going from high-risk to low-risk aged ≥40 years = $\lambda_{2}$

Rate of going from low-risk to high-risk aged 18-39 years = $\varphi_{1}$

Rate of going from low-risk to high-risk aged ≥40 years = $\varphi_{2}$

Force of infection for a low-risk non-NGO MSM = $\theta_{1}$

Force of infection for a low-risk NGO MSM = $\theta_{2}$

Force of infection for a high-risk non-NGO MSM = $\theta_{3}$

Force of infection for a high-risk NGO MSM = $\theta_{4}$

Background death rate for those aged 18-39 years = $\mu_{1}$

Background death rate for those aged ≥40 years = $\mu_{2}$

AIDS-related death rate = $\mu_{3}$

Rate of aging from the 18-39-year group to the ≥40-year group = $a_{1}$

Ratio of reduction in progression if on ART versus not on ART = ψ

Odds ratio of joining NGOs if HIV-positive versus HIV-negative = $\varsigma$

HIV diagnosis rate = 𝜕

Rate of going from acute to chronic = 𝛾

Rate of going from chronic to pre-AIDS = 𝜐

Rate of going from pre-AIDS to AIDS = 𝜀

Rate of starting ART = 𝜋

Rate of stopping ART = 𝜒

**Age 18-39, low-risk, non-NGO MSM**

(1) $\frac{dS_{1}}{dt}=R\left( t \right)\rho_{1}+\tau S_{3}+ \lambda_{1}S_{5}-(\theta_{1}+\mu_{1}+a_{1}+\omega+\varphi_{1})S_{1}$

(2) $\frac{d{EU}_{1}}{dt}=\tau{EU}_{3}+ \lambda_{1}{EU}_{5}+\theta_{1}S_{1}-(\mu_{1}+a_{1}+\omega+\partial+\gamma+\varphi_{1}){EU}_{1}$

(3) $\frac{d{ED}_{1}}{dt}=\tau{ED}_{3}+ \lambda_{1}{ED}_{5}+\partial{EU}_{1}-(\mu_{1}+a_{1}+\omega+\gamma+\varphi_{1}){ED}_{1}$

(4) $\frac{d{CU}_{1}}{dt}=\tau{CU}_{3}+ \lambda_{1}{CU}_{5}+\gamma{EU}_{1}-(\mu_{1}+a_{1}+\omega+\partial+\upsilon+\varphi_{1}){CU}_{1}$

(5) $\frac{d{CD}_{1}}{dt}=\tau{CD}_{3}+ \lambda_{1}{CD}_{5}+\gamma{ED}_{1}+\partial{CU}_{1}+\chi{CA}_{1}-(\mu_{1}+a_{1}+\varsigma\omega+\pi+\upsilon+\varphi_{1}){CD}_{1}$

(6) $\frac{d{CA}_{1}}{dt}=\tau{CA}_{3}+ \lambda_{1}{CA}_{5}+\pi{CD}_{1}-(\mu_{1}+a_{1}+\varsigma\omega+\chi+\psi\upsilon+\varphi_{1}){CA}_{1}$

(7) $\frac{d{PAU}_{1}}{dt}=\tau{PAU}_{3}+ \lambda_{1}{PAU}_{5}+\upsilon{CU}_{1}-(\mu_{1}+a_{1}+\omega+\partial+\varepsilon+\varphi_{1}){PAU}_{1}$

(8) $\frac{d{PAD}_{1}}{dt}=\tau{PAD}_{3}+ \lambda_{1}{PAD}_{5}+\upsilon{CD}_{1}+\partial{PAU}_{1}+\chi{PAA}_{1}-(\mu_{1}+a_{1}+\varsigma\omega+\varepsilon+\pi+\varphi_{1}){PAD}_{1}$

(9) $\frac{d{PAA}_{1}}{dt}=\tau{PAA}_{3}+ \lambda_{1}{PAA}_{5}+\psi\upsilon{CA}_{1}+\pi{PAD}_{1}-(\mu_{1}+a_{1}+\varsigma\omega+\psi\varepsilon+\chi+\varphi_{1}){PAA}_{1}$

(10) $\frac{d{AU}_{1}}{dt}=\tau{AU}_{3}+ \lambda_{1}{AU}_{5}+\varepsilon{PAU}_{1}-(\mu_{1}+a_{1}+\omega+\mu_{3}+\partial+\varphi_{1}){AU}_{1}$

(11) $\frac{d{AD}_{1}}{dt}=\tau{AD}_{3}+ \lambda_{1}{AD}_{5}+\varepsilon{PAD}_{1}+\partial{AU}_{1}+\chi{AA}_{1}-(\mu_{1}+a_{1}+\varsigma\omega+\mu_{3}+\pi+\varphi_{1}){AD}_{1}$

(12) $\frac{d{AA}_{1}}{dt}=\tau{AA}_{3}+ \lambda_{1}{AA}_{5}+\psi\varepsilon{PAA}_{1}+\pi{AD}_{1}-(\mu_{1}+a_{1}+\varsigma\omega+{\psi\mu}_{3}+\chi+\varphi_{1}){AA}_{1}$

(13) $\frac{dD_{1}}{dt}=\mu_{3}\left( {AU}_{1}+{AD}_{1} \right)+\psi\mu_{3}{AA}_{1}$

**Age ≥40, low-risk, non-NGO MSM**

(14) $\frac{dS_{2}}{dt}=a_{1}S_{1}+\tau S_{4}+\lambda_{2}S_{6}-(\theta_{1}+\mu_{2}+\omega+\varphi_{2})S_{2}$

(15) $\frac{d{EU}_{2}}{dt}=a_{1}{EU}_{1}+\tau{EU}_{4}+\lambda_{2}{EU}_{6}+\theta_{1}S_{2}-(\mu_{2}+\omega+\partial+\gamma+\varphi_{2}){EU}_{2}$

(16) $\frac{d{ED}_{2}}{dt}=a_{1}{ED}_{1}+\tau{ED}_{4}+\lambda_{2}{ED}_{6}+\partial{EU}_{2}-(\mu_{2}+\omega+\gamma+\varphi_{2}){ED}_{2}$

(17) $\frac{d{CU}_{2}}{dt}=a_{1}{CU}_{1}+\tau{CU}_{4}+\lambda_{2}{CU}_{6}+\gamma{EU}_{2}-(\mu_{2}+\omega+\partial+\upsilon+\varphi_{2}){CU}_{2}$

(18) $\frac{d{CD}_{2}}{dt}=a_{1}{CD}_{1}+\tau{CD}_{4}+\lambda_{2}{CD}_{6}+\gamma{CD}_{2}+\partial{CU}_{2}+\chi{CA}_{2}-(\mu_{2}+\varsigma\omega+\pi+\upsilon+\varphi_{2}){CD}_{2}$

(19) $\frac{d{CA}_{2}}{dt}=a_{1}{CA}_{1}+\tau{CA}_{4}+\lambda_{2}{CA}_{6}+\pi{CD}_{2}-(\mu_{2}+\varsigma\omega+\chi+\psi\upsilon+\varphi_{2}){CA}_{2}$

(20) $\frac{d{PAU}_{2}}{dt}=a_{1}{PAU}_{1}+\tau{PAU}_{4}+\lambda_{2}{PAU}_{6}+\upsilon{CU}_{2}-(\mu_{2}+\omega+\partial+\varepsilon+\varphi_{2}){PAU}_{2}$

(21) $\frac{d{PAD}_{2}}{dt}=a_{1}{PAD}_{1}+\tau{PAD}_{4}+\lambda_{2}{PAD}_{6}+\upsilon{CD}_{2}+\partial{PAU}_{2}+\chi{PAA}_{2}-(\mu_{2}+\varsigma\omega+\varepsilon+\pi+\varphi_{2}){PAD}_{2}$

(22) $\frac{d{PAA}_{2}}{dt}=a_{1}{PAA}_{1}+\tau{PAA}_{4}+\lambda_{2}{PAA}_{6}+\psi\upsilon{CA}_{2}+\pi{PAD}_{2}-(\mu_{2}+\varsigma\omega+\psi\varepsilon+\chi+\varphi_{2}){PAA}_{2}$

(23) $\frac{d{AU}_{2}}{dt}=a_{1}{AU}_{1}+\tau{AU}_{4}+\lambda_{2}{AU}_{6}+\varepsilon{PAU}_{2}-(\mu_{2}+\omega+\mu_{3}+\partial+\varphi_{2}){AU}_{2}$

(24) $\frac{d{AD}_{2}}{dt}=a_{1}{AD}_{1}+\tau{AD}_{4}+\lambda_{2}{AD}_{6}+\varepsilon{PAD}_{2}+\partial{AU}_{2}+\chi{AA}_{2}-(\mu_{2}+\varsigma\omega+\mu_{3}+\pi+\varphi_{2}){AD}_{2}$

(25) $\frac{d{AA}_{2}}{dt}=a_{1}{AA}_{1}+\tau{AA}_{4}+\lambda_{2}{AA}_{6}+\psi\varepsilon{PAA}_{2}+\pi{AD}_{2}-(\mu_{2}+\varsigma\omega+{\psi\mu}_{3}+\chi+\varphi_{2}){AA}_{2}$

(26) $\frac{dD_{2}}{dt}=\mu_{3}\left( {AU}_{2}+{AD}_{2} \right)+\psi\mu_{3}{AA}_{2}$

**Age 18-39, low-risk, NGO MSM**

(27) $\frac{dS_{3}}{dt}=\omega S_{1}+\lambda_{1}S_{7}-(\theta_{2}+\mu_{1}+a_{1}+\tau+\varphi_{1})S_{3}$

(28) $\frac{d{EU}_{3}}{dt}=\omega{EU}_{1}+\lambda_{1}{EU}_{7}+\theta_{2}S_{3}-(\mu_{1}+a_{1}+\tau+\partial+\gamma+\varphi_{1}){EU}_{3}$

(29) $\frac{d{ED}_{3}}{dt}=\omega{ED}_{1}+\lambda_{1}{ED}_{7}+\partial{EU}_{3}-(\mu_{1}+a_{1}+\tau+\gamma+\varphi_{1}){ED}_{3}$

(30) $\frac{d{CU}_{3}}{dt}=\omega{CU}_{1}+\lambda_{1}{CU}_{7}+\gamma{EU}_{3}-(\mu_{1}+a_{1}+\tau+\partial+\upsilon+\varphi_{1}){CU}_{3}$

(31) $\frac{d{CD}_{3}}{dt}=\varsigma\omega{CD}_{1}+\lambda_{1}{CD}_{7}+\gamma{ED}_{3}+\partial{CU}_{3}+\chi{CA}_{3}-(\mu_{1}+a_{1}+\tau+\pi+\upsilon+\varphi_{1}){CD}_{3}$

(32) $\frac{d{CA}_{3}}{dt}=\varsigma\omega{CA}_{1}+\lambda_{1}{CA}_{7}+\pi{CD}_{3}-(\mu_{1}+a_{1}+\tau+\chi+\psi\upsilon+\varphi_{1}){CA}_{3}$

(33) $\frac{d{PAU}_{3}}{dt}=\omega{PAU}_{1}+\lambda_{1}{PAU}_{7}+\upsilon{CU}_{3}-(\mu_{1}+a_{1}+\tau+\partial+\varepsilon+\varphi_{1}){PAU}_{3}$

(34) $\frac{d{PAD}_{3}}{dt}=\varsigma\omega{PAD}_{1}+\lambda_{1}{PAD}_{7}+\upsilon{CD}_{3}+\partial{PAU}_{3}+\chi{PAA}_{3}-(\mu_{1}+a_{1}+\tau+\varepsilon+\pi+\varphi_{1}){PAD}_{3}$

(35) $\frac{d{PAA}_{3}}{dt}=\varsigma\omega{PAA}_{1}+\lambda_{1}{PAA}_{7}+\psi\upsilon{CA}_{3}+\pi{PAD}_{3}-(\mu_{1}+a_{1}+\tau+\psi\varepsilon+\chi+\varphi_{1}){PAA}_{3}$

(36) $\frac{d{AU}_{3}}{dt}=\omega{AU}_{1}+\lambda_{1}{AU}_{7}+\varepsilon{PAU}_{3}-(\mu_{1}+a_{1}+\tau+\mu_{3}+\partial+\varphi_{1}){AU}_{3}$

(37) $\frac{d{AD}_{3}}{dt}=\varsigma\omega{AD}_{1}+\lambda_{1}{AD}_{7}+\varepsilon{PAD}_{3}+\partial{AU}_{3}+\chi{AA}_{3}-(\mu_{1}+a_{1}+\tau+\mu_{3}+\pi+\varphi_{1}){AD}_{3}$

(38) $\frac{d{AA}_{3}}{dt}=\varsigma\omega{AA}_{1}+\lambda_{1}{AA}_{7}+\psi\varepsilon{PAA}_{3}+\pi{AD}_{3}-(\mu_{1}+a_{1}+\tau+{\psi\mu}_{3}+\chi+\varphi_{1}){AA}_{3}$

(39) $\frac{dD_{3}}{dt}=\mu_{3}\left( {AU}_{3}+{AD}_{3} \right)+\psi\mu_{3}{AA}_{3}$

**Age ≥40, low-risk, NGO MSM**

(40) $\frac{dS_{4}}{dt}=a_{1}S_{3}+\omega S_{2}+\lambda_{2}S_{8}-(\theta_{2}+\mu_{2}+\tau+\varphi_{2})S_{4}$

(41) $\frac{d{EU}_{4}}{dt}=a_{1}{EU}_{3}+\omega{EU}_{2}+\lambda_{2}{EU}_{8}+\theta_{2}S_{4}-(\mu_{2}+\tau+\partial+\gamma+\varphi_{2}){EU}_{4}$

(42) $\frac{d{ED}_{4}}{dt}=a_{1}{ED}_{3}+ \omega{ED}_{2}+\lambda_{2}{ED}_{8}+\partial{EU}_{4}-(\mu_{2}+\tau+\gamma+\varphi_{2}){ED}_{4}$

(43) $\frac{d{CU}_{4}}{dt}=a_{1}{CU}_{3}+\omega{CU}_{2}+\lambda_{2}{CU}_{8}+\gamma{EU}_{4}-(\mu_{2}+\tau+\partial+\upsilon+\varphi_{2}){CU}_{4}$

(44) $\frac{d{CD}_{4}}{dt}=a_{1}{CD}_{3}+\varsigma\omega{CD}_{2}+\lambda_{2}{CD}_{8}+\gamma{CD}_{4}+\partial{CU}_{4}+\chi{CA}_{4}-(\mu_{2}+\tau+\pi+\upsilon+\varphi_{2}){CD}_{4}$

(45) $\frac{d{CA}_{4}}{dt}=a_{1}{CA}_{3}+\varsigma\omega{CA}_{2}+\lambda_{2}{CA}_{8}+\pi{CD}_{2}-(\mu_{2}+\tau+\chi+\psi\upsilon+\varphi_{2}){CA}_{4}$

(46) $\frac{d{PAU}_{4}}{dt}=a_{1}{PAU}_{3}+\omega{PAU}_{2}+\lambda_{2}{PAU}_{8}+\upsilon{CU}_{4}-(\mu_{2}+\tau+\partial+\varepsilon+\varphi_{2}){PAU}_{4}$

(47) $\frac{d{PAD}_{4}}{dt}=a_{1}{PAD}_{3}+\varsigma\omega{PAD}_{2}+\lambda_{2}{PAD}_{8}+\upsilon{CD}_{4}+\partial{PAU}_{4}+\chi{PAA}_{4}-(\mu_{2}+\tau+\varepsilon+\pi+\varphi_{2}){PAD}_{4}$

(48) $\frac{d{PAA}_{4}}{dt}=a_{1}{PAA}_{3}+\varsigma\omega{PAA}_{2}+\lambda_{2}{PAA}_{8}+\psi\upsilon{CA}_{4}+\pi{PAD}_{4}-(\mu_{2}+\tau+\psi\varepsilon+\chi+\varphi_{2}){PAA}_{4}$

(49) $\frac{d{AU}_{4}}{dt}=a_{1}{AU}_{3}+\omega{AU}_{2}+\lambda_{2}{AU}_{8}+\varepsilon{PAU}_{4}-(\mu_{2}+\tau+\mu_{3}+\partial+\varphi_{2}){AU}_{4}$

(50) $\frac{d{AD}_{4}}{dt}=a_{1}{AD}_{3}+ \varsigma\omega{AD}_{2}+\lambda_{2}{AD}_{8}+\varepsilon{PAD}_{4}+\partial{AU}_{4}+\chi{AA}_{4}-(\mu_{2}+\tau+\mu_{3}+\pi+\varphi_{2}){AD}_{4}$

(51) $\frac{d{AA}_{4}}{dt}=a_{1}{AA}_{3}+\varsigma\omega{AA}_{2}+\lambda_{2}{AA}_{8}+\psi\varepsilon{PAA}_{4}+\pi{AD}_{4}-(\mu_{2}+\tau+{\psi\mu}_{3}+\chi+\varphi_{2}){AA}_{4}$

(52) $\frac{dD_{4}}{dt}=\mu_{3}\left( {AU}_{4}+{AD}_{4} \right)+\psi\mu_{3}{AA}_{4}$

**Age 18-39, high-risk, non-NGO MSM**

(53) $\frac{dS_{5}}{dt}= R\left( t \right)\rho_{2}+\tau S_{7}+ \lambda_{1}S_{1}-(\theta_{3}+\mu_{1}+a_{1}+\omega+\varphi_{3})S_{5}$

(54) $\frac{d{EU}_{5}}{dt}=\tau{EU}_{7}+ \varphi_{1}{EU}_{1}+\theta_{3}S_{5}-(\mu_{1}+a_{1}+\omega+\partial+\gamma+\varphi_{3}){EU}_{5}$

(55) $\frac{d{ED}_{5}}{dt}=\tau{ED}_{7}+ \varphi_{1}{ED}_{1}+\partial{EU}_{5}-(\mu_{1}+a_{1}+\omega+\gamma+\lambda_{1}){ED}_{5}$

(56) $\frac{d{CU}_{5}}{dt}=\tau{CU}_{7}+ \varphi_{1}{CU}_{1}+\gamma{EU}_{5}-(\mu_{1}+a_{1}+\omega+\partial+\upsilon+\lambda_{1}){CU}_{5}$

(57) $\frac{d{CD}_{5}}{dt}=\tau{CD}_{7}+ \varphi_{1}{CD}_{1}+\gamma{ED}_{5}+\partial{CU}_{5}+\chi{CA}_{5}-(\mu_{1}+a_{1}+\varsigma\omega+\pi+\upsilon+\lambda_{1}){CD}_{5}$

(58) $\frac{d{CA}_{5}}{dt}=\tau{CA}_{7}+ \varphi_{1}{CA}_{1}+\pi{CD}_{5}-(\mu_{1}+a_{1}+\varsigma\omega+\chi+\psi\upsilon+\lambda_{1}){CA}_{5}$

(59) $\frac{d{PAU}_{5}}{dt}=\tau{PAU}_{7}+ \varphi_{1}{PAU}_{1}+\upsilon{CU}_{5}-(\mu_{1}+a_{1}+\omega+\partial+\varepsilon+\lambda_{1}){PAU}_{5}$

(60) $\frac{d{PAD}_{5}}{dt}=\tau{PAD}_{7}+ \varphi_{1}{PAD}_{1}+\upsilon{CD}_{5}+\partial{PAU}_{5}+\chi{PAA}_{5}-(\mu_{1}+a_{1}+\varsigma\omega+\varepsilon+\pi+\lambda_{1}){PAD}_{5}$

(61) $\frac{d{PAA}_{5}}{dt}=\tau{PAA}_{7}+ \varphi_{1}{PAA}_{1}+\psi\upsilon{CA}_{5}+\pi{PAD}_{5}-(\mu_{1}+a_{1}+\varsigma\omega+\psi\varepsilon+\chi+\lambda_{1}){PAA}_{5}$

(62) $\frac{d{AU}_{5}}{dt}=\tau{AU}_{7}+ \varphi_{1}{AU}_{1}+\varepsilon{PAU}_{5}-(\mu_{1}+a_{1}+\omega+\mu_{3}+\partial+\lambda_{1}){AU}_{5}$

(63) $\frac{d{AD}_{5}}{dt}=\tau{AD}_{7}+ \varphi_{1}{AD}_{1}+\varepsilon{PAD}_{5}+\partial{AU}_{5}+\chi{AA}_{5}-(\mu_{1}+a_{1}+\varsigma\omega+\mu_{3}+\pi+\lambda_{1}){AD}_{5}$

(64) $\frac{d{AA}_{5}}{dt}=\tau{AA}_{7}+ \varphi_{1}{AA}_{1}+\psi\varepsilon{PAA}_{5}+\pi{AD}_{5}-(\mu_{1}+a_{1}+\varsigma\omega+{\psi\mu}_{3}+\chi+\lambda_{1}){AA}_{5}$

(65) $\frac{dD_{5}}{dt}=\mu_{3}\left( {AU}_{5}+{AD}_{5} \right)+\psi\mu_{3}{AA}_{5}$

**Age ≥40, high-risk, non-NGO MSM**

(66) $\frac{dS_{6}}{dt}=a_{1}S_{5}+\tau S_{8}+\varphi_{2}S_{2}-(\theta_{3}+\mu_{2}+\omega+\lambda_{2})S_{6}$

(67) $\frac{d{EU}_{6}}{dt}=a_{1}{EU}_{5}+\tau{EU}_{8}+\varphi_{2}{EU}_{2}+\theta_{3}S_{6}-(\mu_{2}+\omega+\partial+\gamma+\lambda_{2}){EU}_{6}$

(68) $\frac{d{ED}_{6}}{dt}=a_{1}{ED}_{5}+\tau{ED}_{8}+\varphi_{2}{ED}_{2}+\partial{EU}_{6}-(\mu_{2}+\omega+\gamma+\lambda_{2}){ED}_{6}$

(69) $\frac{d{CU}_{6}}{dt}=a_{1}{CU}_{5}+\tau{CU}_{8}+\varphi_{2}{CU}_{2}+\gamma{EU}_{6}-(\mu_{2}+\omega+\partial+\upsilon+\lambda_{2}){CU}_{6}$

(70) $\frac{d{CD}_{6}}{dt}=a_{1}{CD}_{5}+\tau{CD}_{8}+\varphi_{2}{CD}_{2}+\gamma{CD}_{6}+\partial{CU}_{6}+\chi{CA}_{6}-(\mu_{2}+\varsigma\omega+\pi+\upsilon+\lambda_{2}){CD}_{6}$

(71) $\frac{d{CA}_{6}}{dt}=a_{1}{CA}_{5}+\tau{CA}_{8}+\varphi_{2}{CA}_{2}+\pi{CD}_{6}-(\mu_{2}+\varsigma\omega+\chi+\psi\upsilon+\lambda_{2}){CA}_{6}$

(72) $\frac{d{PAU}_{6}}{dt}=a_{1}{PAU}_{5}+\tau{PAU}_{8}+\varphi_{2}{PAU}_{2}+\upsilon{CU}_{6}-(\mu_{2}+\omega+\partial+\varepsilon+\lambda_{2}){PAU}_{6}$

(73) $\frac{d{PAD}_{6}}{dt}=a_{1}{PAD}_{5}+\tau{PAD}_{8}+\varphi_{2}{PAD}_{2}+\upsilon{CD}_{6}+\partial{PAU}_{6}+\chi{PAA}_{6}-(\mu_{2}+\varsigma\omega+\varepsilon+\pi+\lambda_{2}){PAD}_{6}$

(74) $\frac{d{PAA}_{6}}{dt}=a_{1}{PAA}_{5}+\tau{PAA}_{8}+\varphi_{2}{PAA}_{2}+\psi\upsilon{CA}_{6}+\pi{PAD}_{6}-(\mu_{2}+\varsigma\omega+\psi\varepsilon+\chi+\lambda_{2}){PAA}_{6}$

(75) $\frac{d{AU}_{6}}{dt}=a_{1}{AU}_{5}+\tau{AU}_{8}+\varphi_{2}{AU}_{2}+\varepsilon{PAU}_{6}-(\mu_{2}+\omega+\mu_{3}+\partial+\lambda_{2}){AU}_{6}$

(76) $\frac{d{AD}_{6}}{dt}=a_{1}{AD}_{5}+\tau{AD}_{8}+\varphi_{2}{AD}_{2}+\varepsilon{PAD}_{6}+\partial{AU}_{6}+\chi{AA}_{6}-(\mu_{2}+\varsigma\omega+\mu_{3}+\pi+\lambda_{2}){AD}_{6}$

(77) $\frac{d{AA}_{6}}{dt}=a_{1}{AA}_{5}+\tau{AA}_{8}+\varphi_{2}{AA}_{2}+\psi\varepsilon{PAA}_{6}+\pi{AD}_{6}-(\mu_{2}+\varsigma\omega+{\psi\mu}_{3}+\chi+\lambda_{2}){AA}_{6}$

(78) $\frac{dD_{6}}{dt}=\mu_{3}\left( {AU}_{6}+{AD}_{6} \right)+\psi\mu_{3}{AA}_{6}$

**Age 18-39, high-risk, NGO MSM**

(79) $\frac{dS_{7}}{dt}=\omega S_{5}+\varphi_{1}S_{3}-(\theta_{4}+\mu_{1}+a_{1}+\tau+\lambda_{1})S_{7}$

(80) $\frac{d{EU}_{7}}{dt}=\omega{EU}_{5}+\varphi_{1}{EU}_{3}+\theta_{4}S_{7}-(\mu_{1}+a_{1}+\tau+\partial+\gamma+\lambda_{1}){EU}_{7}$

(81) $\frac{d{ED}_{7}}{dt}=\omega{ED}_{5}+\varphi_{1}{ED}_{3}+\partial{EU}_{7}-(\mu_{1}+a_{1}+\tau+\gamma+\lambda_{1}){ED}_{7}$

(82) $\frac{d{CU}_{7}}{dt}=\omega{CU}_{5}+\varphi_{1}{CU}_{3}+\gamma{EU}_{7}-(\mu_{1}+a_{1}+\tau+\partial+\upsilon+\lambda_{1}){CU}_{7}$

(83) $\frac{d{CD}_{7}}{dt}=\varsigma\omega{CD}_{5}+\varphi_{1}{CD}_{3}+\gamma{ED}_{7}+\partial{CU}_{7}+\chi{CA}_{7}-(\mu_{1}+a_{1}+\tau+\pi+\upsilon+\lambda_{1}){CD}_{7}$

(84) $\frac{d{CA}_{7}}{dt}=\varsigma\omega{CA}_{5}+\varphi_{1}{CA}_{3}+\pi{CD}_{7}-(\mu_{1}+a_{1}+\tau+\chi+\psi\upsilon+\lambda_{1}){CA}_{7}$

(85) $\frac{d{PAU}_{7}}{dt}=\omega{PAU}_{5}+\varphi_{1}{PAU}_{3}+\upsilon{CU}_{7}-(\mu_{1}+a_{1}+\tau+\partial+\varepsilon+\lambda_{1}){PAU}_{7}$

(86) $\frac{d{PAD}_{7}}{dt}=\varsigma\omega{PAD}_{5}+\varphi_{1}{PAD}_{3}+\upsilon{CD}_{7}+\partial{PAU}_{7}+\chi{PAA}_{7}-(\mu_{1}+a_{1}+\tau+\varepsilon+\pi+\lambda_{1}){PAD}_{7}$

(87) $\frac{d{PAA}_{7}}{dt}=\varsigma\omega{PAA}_{5}+\varphi_{1}{PAA}_{3}+\psi\upsilon{CA}_{7}+\pi{PAD}_{7}-(\mu_{1}+a_{1}+\tau+\psi\varepsilon+\chi+\lambda_{1}){PAA}_{7}$

(88) $\frac{d{AU}_{7}}{dt}=\omega{AU}_{5}+\varphi_{1}{AU}_{3}+\varepsilon{PAU}_{7}-(\mu_{1}+a_{1}+\tau+\mu_{3}+\partial+\lambda_{1}){AU}_{7}$

(89) $\frac{d{AD}_{7}}{dt}=\varsigma\omega{AD}_{5}+\varphi_{1}{AD}_{3}+\varepsilon{PAD}_{7}+\partial{AU}_{7}+\chi{AA}_{7}-(\mu_{1}+a_{1}+\tau+\mu_{3}+\pi+\lambda_{1}){AD}_{7}$

(90) $\frac{d{AA}_{7}}{dt}=\varsigma\omega{AA}_{5}+\varphi_{1}{AA}_{3}+\psi\varepsilon{PAA}_{7}+\pi{AD}_{7}-(\mu_{1}+a_{1}+\tau+{\psi\mu}_{3}+\chi+\lambda_{1}){AA}_{7}$

(91) $\frac{dD_{7}}{dt}=\mu_{3}\left( {AU}_{7}+{AD}_{7} \right)+\psi\mu_{3}{AA}_{7}$

**Age ≥40, high-risk, NGO MSM**

(92) $\frac{dS_{8}}{dt}=a_{1}S_{7}+\omega S_{6}+\varphi_{2}S_{4}-(\theta_{4}+\mu_{2}+\tau+\lambda_{2})S_{8}$

(93) $\frac{d{EU}_{8}}{dt}=a_{1}{EU}_{7}+\omega{EU}_{6}+\varphi_{2}{EU}_{4}+\theta_{4}S_{8}-(\mu_{2}+\tau+\partial+\gamma+\lambda_{2}){EU}_{8}$

(94) $\frac{d{ED}_{8}}{dt}=a_{1}{ED}_{7}+ \omega{ED}_{6}+\varphi_{2}{ED}_{4}+\partial{EU}_{8}-(\mu_{2}+\tau+\gamma+\lambda_{2}){ED}_{8}$

(95) $\frac{d{CU}_{8}}{dt}=a_{1}{CU}_{7}+\omega{CU}_{6}+\varphi_{2}{CU}_{4}+\gamma{EU}_{8}-(\mu_{2}+\tau+\partial+\upsilon+\lambda_{2}){CU}_{8}$

(96) $\frac{d{CD}_{8}}{dt}=a_{1}{CD}_{7}+\varsigma\omega{CD}_{6}+\varphi_{2}{CD}_{4}+\gamma{CD}_{8}+\partial{CU}_{8}+\chi{CA}_{8}-(\mu_{2}+\tau+\pi+\upsilon+\lambda_{2}){CD}_{8}$

(97) $\frac{d{CA}_{8}}{dt}=a_{1}{CA}_{7}+\varsigma\omega{CA}_{6}+\varphi_{2}{CA}_{4}+\pi{CD}_{8}-(\mu_{2}+\tau+\chi+\psi\upsilon+\lambda_{2}){CA}_{8}$

(98) $\frac{d{PAU}_{8}}{dt}=a_{1}{PAU}_{7}+\omega{PAU}_{6}+\varphi_{2}{PAU}_{4}+\upsilon{CU}_{8}-(\mu_{2}+\tau+\partial+\varepsilon+\lambda_{2}){PAU}_{8}$

(99) $\frac{d{PAD}_{8}}{dt}=a_{1}{PAD}_{7}+\varsigma\omega{PAD}_{6}+\varphi_{2}{PAD}_{4}+\upsilon{CD}_{8}+\partial{PAU}_{8}+\chi{PAA}_{8}-(\mu_{2}+\tau+\varepsilon+\pi+\lambda_{2}){PAD}_{8}$

(100) $\frac{d{PAA}_{8}}{dt}=a_{1}{PAA}_{7}+\varsigma\omega{PAA}_{6}+\varphi_{2}{PAA}_{4}+\psi\upsilon{CA}_{8}+\pi{PAD}_{8}-(\mu_{2}+\tau+\psi\varepsilon+\chi+\lambda_{2}){PAA}_{8}$

(101) $\frac{d{AU}_{8}}{dt}=a_{1}{AU}_{7}+\omega{AU}_{6}+\varphi_{2}{AU}_{4}+\varepsilon{PAU}_{8}-(\mu_{2}+\tau+\mu_{3}+\partial+\lambda_{2}){AU}_{8}$

(102) $\frac{d{AD}_{8}}{dt}=a_{1}{AD}_{7}+ \varsigma\omega{AD}_{6}+\varphi_{2}{AD}_{4}+\varepsilon{PAD}_{8}+\partial{AU}_{8}+\chi{AA}_{8}-(\mu_{2}+\tau+\mu_{3}+\pi+\lambda_{2}){AD}_{8}$

(103) $\frac{d{AA}_{8}}{dt}=a_{1}{AA}_{7}+\varsigma\omega{AA}_{6}+\varphi_{2}{AA}_{4}+\psi\varepsilon{PAA}_{8}+\pi{AD}_{8}-(\mu_{2}+\tau+{\psi\mu}_{3}+\chi+\lambda_{2}){AA}_{4}$

(104) $\frac{dD_{8}}{dt}=\mu_{3}\left( {AU}_{8}+{AD}_{8} \right)+\psi\mu_{3}{AA}_{8}$

**Supplementary figure 1:** Model schematics of how MSM transition through (a) age, low/high-risk sexual behaviour, and NGO status groups; b) different stages of HIV progression†.

a)


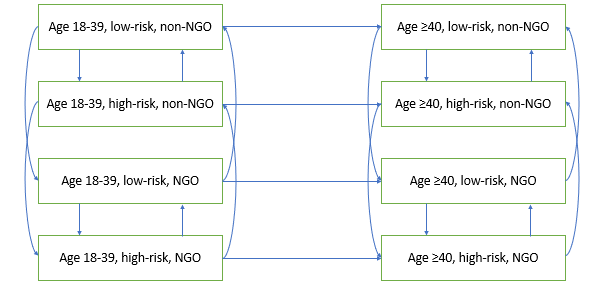


b)


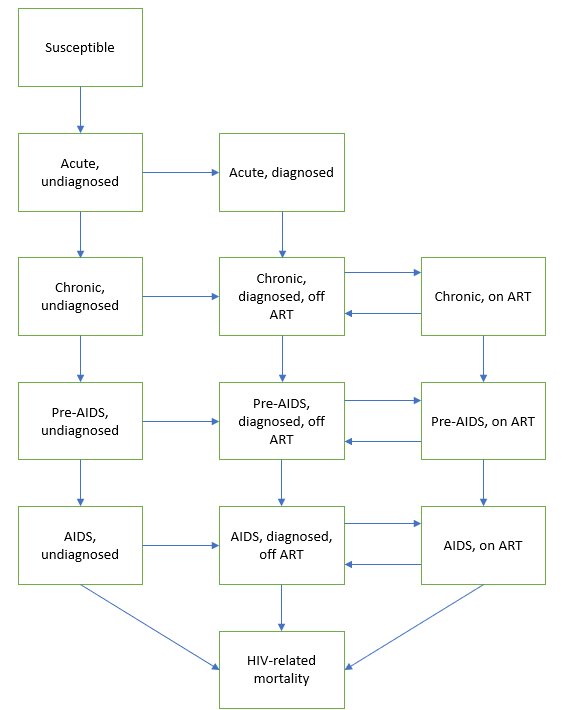


MSM: Men who have sex with men. NGO: Non-governmental organisation. PrEP: Pre-exposure Prophylaxis. ART: Antiretroviral therapy.

**Supplementary figure 2:** Data and model projections of the number of MSM that are NGO clients (status quo projections)

MSM: men who have sex with men. NGO: Non-governmental organisation. 95%CrI: 95% credibility interval. APH: Alliance for Public Health, Ukraine.

**Supplementary figure 3:** A comparison of data with our modelled HIV prevalence projections stratified by low/high risk group and NGO status for MSM aged 18-39 years. Whiskers on the data points denote the 95% confidence intervals. Black line gives median model projections and blue shading is the 95% interval of the model projections.

NGO: Non-governmental Organisation. MSM: Men who have sex with men. IBBS: Integrated Bio-Behavioural Survey. High-risk defined as 10 or more sexual acts in a month.

**Supplementary figure 4:** The proportion of HIV-positive MSM aged 18-39 that are on ART stratified by NGO client status.

MSM: men who have sex with men. ART: antiretroviral therapy. NGO: Non-governmental organisation. IBBS: Integrated Bio-Behavioural Survey. 95%CrI: 95% credibility interval.

**Supplementary figure 5:** The proportion of HIV-positive MSM that have been diagnosed with HIV, and the proportion of HIV-positive MSM that are on ART, for various scenarios from 1990-2030.

MSM: men who have sex with men. ART: antiretroviral therapy. NGO: Non-governmental organisation. 95%CrI: 95% credibility interval.

**Investigating assumptions about NGO effectiveness**

In our previous paper(13), we found that being an NGO client (versus not) was associated with reducing various risk behaviours and increasing testing and linkage to care. One potential explanation for the differences seen between MSM in contact with NGOs and those that are not, is that people diagnosed with HIV will alter their behaviours. We found differences in recently buying or receiving condoms between HIV- and HIV+ MSM (77% vs 90%, p<0.001) and in condom use at the last anal sex (73% vs 80%, p=0.002). We expanded on our previous analyses, to investigate whether associations for condom use held up when stratifying by HIV status (testing and linkage to care would not be applicable for HIV+ and HIV- MSM, respectively). We found that being an NGO client (versus not) was associated with heightened buying/receiving condoms and condom use for both the HIV-positive and HIV-negative MSM, although the adjusted odds ratios differed between the two populations.

- Condom used last anal sex: HIV- 1.22 (1.13-1.32) & HIV+ 2.18 (1.65-2.88)
- Condom used last anal sex with permanent partner: HIV- 1.19 (1.09-1.31) & HIV+ 1.98 (1.42-2.76)
- Condom used last anal sex with casual partner: HIV- 1.89 (1.63-2.20) & HIV+ 4.67 (2.49-8.77)
- Recently buying/receiving condoms: HIV- 22.01 (18.72-25.87) & HIV+ 10.95 (6.93-17.21)

Another potential explanation is that MSM in contact with NGOs are more likely to be those at higher risk of contracting HIV and alter their behaviours accordingly. In the IBBS data there was a statistical difference (p=0.001) in the proportion of high-risk MSM (those with 10 or more sexual contacts in the past month) that were NGO clients or not, however, the actual difference was not that large: 23% vs 21%, respectively. Additionally, in analyses where we examined behaviours between MSM self-reporting as HIV-negative that were classified as high-risk and not high-risk, the high-risk MSM were more likely to have received a HIV test in the last year (47% vs 43%, p<0.001), however, they were less likely to have used condoms at their last anal intercourse (64% vs 76%, p<0.001). Together this indicates that the large differences in HIV outcomes between MSM in contact with NGOs or not, is unlikely to be entirely explained by differences in behaviours between MSM considered high-risk for HIV or not.

Regarding rapid HIV testing in Ukraine, there are other venues where it may be performed besides NGOs. These venues include mobile clinics/on the street or at home, AIDS centers, general hospitals, confidence cabinets, private labs, private clinics, and self-testing by rapid tests bought in pharmacies. In the IBBS data, 43% of MSM had received a HIV test at NGOs, a figure that varied from 18% of non-NGO clients to 72% of NGO clients. This shows the outsized role NGOs have in providing HIV testing for MSM in Ukraine, far above their levels of coverage (around 28% of MSM).

In the table below, we show the results where the status quo NGO scenario is compared to a scenario where NGOs are 50% less effective regarding testing, linking people to antiretroviral treatment, and condom distribution, which could be interpreted as 50% of the effects we are currently prescribing to NGOs happening due to other causes.

**Supplementary table 3:** Model results for selected scenarios over different time periods, compared to a scenario where NGOs are 50% less effective – median (95% credibility intervals).

|  | **Analysis period: 2016-2020** | | |
| --- | --- | --- | --- |
| **Outcome** | **Status quo** | **50% effect of NGOs 2016-2020** | **No NGOs 2016-2020** |
| HIV infections during period | 6151 (5133, 7008) | 6787 (5683, 7722) | 7633 (6411, 8802) |
| Percentage difference vs status quo | NA | 10% (9%, 12%) | 25% (21%, 30%) |
| MSM HIV incidence (per 100py) at end of period | 0.73 (0.59, 0.85) | 0.85 (0.69, 1.01) | 1.05 (0.84, 1.27) |
| Percentage difference vs status quo | NA | 17% (15%, 21%) | 44% (36%, 59%) |
| MSM HIV prevalence at end of period | 6.2% (5.3%, 7.0%) | 6.5% (5.6%, 7.3%) | 6.9% (5.9%, 7.7%) |
| Percentage difference vs status quo | NA | 4% (3%, 5%) | 10% (9%, 12%) |
| MSM HIV deaths during period | 6253 (5587, 6954) | 6479 (5786, 7182) | 6822 (6065, 7580) |
| Percentage difference vs status quo | NA | 4% (3%, 4%) | 9% (7%, 12%) |

NGO: Non-governmental organisation. MSM: men who have sex with men.

In table 3 in the main manuscript, we show that the status quo scenario is still cost-effective at the 0.5xGDP level when compared with a scenario where 50% of the effects of NGOs are prescribed to other causes (cost assumptions are explained on the next page).

**Cost and health utility assumptions**

Unit cost estimates for ART and NGO services for MSM (HIV counselling and testing, condom distribution, HIV case management) came from published reports and APH budget data for Ukraine. For the base case, we used the most recent estimates that provided sufficient cost breakdowns. Ukrainian hryvnia (UAH) were converted to United States dollars (US$) using historical exchange rates(14) and then inflated to 2018 US$ using the World Bank’s consumer price index ratios from Ukraine(15). Unit cost estimates are given in supplementary table 4 below. MSM that were not on ART nor clients of NGOs were assumed to have no cost. Conversely, all NGO clients were assigned an annual basic service package cost (US$17.00-$24.45) based on APH budget data, whilst all HIV-positive MSM on ART were assigned an annual ART cost (US$280.76 to $312.53(16)). If they were both an NGO client and on ART, then they were assigned both costs. Lastly, all MSM newly diagnosed as HIV-positive whilst NGO clients were assigned a one-off cost in the year following diagnosis for case management and psychosocial services (US$82 to $182). Cost estimates were incorporated into the model by assigning them to anyone on ART or that was an NGO client, and anyone newly diagnosed with HIV.

For the comparator scenarios where NGO effects were removed or prescribed to other causes from 2016-20 (or other time periods), the costs assigned to NGOs would be removed and only the non-NGO costs (ART for non-NGO clients) remained.

We assigned disability weights from the Global Burden of Disease study(12) to each stage of HIV in the model to calculate disability adjusted life years (DALYs) for a specific model run.

Uncertainty in costs and disability weights were included through sampling these parameter estimates from the uncertainty distributions in Supplementary Tables 1, 2 and 4, producing 500 sets of costs and disability weights. These were assigned to the 500 baseline model fits.

*Cost-effectiveness sensitivity analyses*

We performed numerous one-way sensitivity analyses to determine the effect of various assumptions on the ICER. These included: incorporating a different annual cost for being an NGO client based on a detailed costing done by Deloitte in 2018(17), which assumes a different cost for HIV-positive (Triangular distribution: US$3.18, $0.95-$6.36 for condoms alone) and HIV-negative NGO clients (Triangular distribution: US$13.21, $8.69-$21.26 for condoms and HIV testing and counselling); changing the end of the time horizon to 2040 (15 years) or 2050 (25 years); changing the discount rate to 0% or 5% per annum (Baseline: 3%); using alternative annual costs for ART (Triangular distribution: US$1,016, $972-$1,082) from the Deloitte costing(18); and assigning different percentages (in 10% increments) of the NGO effects to other causes.

We also calculated the ICER per death averted and per infection averted.

**Supplementary table 4:** Unit costs of antiretroviral therapy (ART) and NGO services provided for MSM in Ukraine, in 2018 US dollars (US$)

| **Unit** | **Cost per patient** | **Comments** |  |
| --- | --- | --- | --- |
| **NGO costs used in base case analysis (APH budget)** | | |  |
| Annual NGO cost for MSM regardless of HIV status | $17 basic service package, alternative estimate of $24.45 which is total cost per client reached. (Uniform range used to sample between two estimates) | Includes counselling, testing, staff costs and HIV navigation, administration costs. Assumes 1.4 rapid HIV tests per year, 0.3 HCV, HBV, and syphilis tests per year, one set of information materials, 30 condoms and 15 lubricants. |  |
| **NGO costs used in sensitivity analysis (17)** | | |  |
| HIV negative or HIV undiagnosed MSM annual NGO cost | $13.21 ($8.69 to $21.26 for HIV counselling and testing plus range of condom costs as below) | Includes cost of condoms and HIV counselling and testing |  |
| Diagnosed HIV positive MSM annual NGO cost | $3.18 (lower bound 30 condoms per year $0.95, upper bound double of estimate: $6.36) | Condoms only (100 per year) |  |
| **ART-related costs used in base case analysis** | | |  |
| One off NGO cost in first year of HIV diagnosis | $132 (triangular distribution $82 to $182) (17) | Cost of case management and psychosocial services for each person |  |
| ART annual cost | $293.47 (triangular distribution $280.76 to $312.53) (16) | Estimate of $276.50 includes drug, staff, and test costs. We added 6% overheads and uncertainty bounds based on ART costs reported elsewhere (18). |  |
| **ART-related costs used in sensitivity analysis (18)** | | |  |
| ART annual cost | $1,016 (triangular distribution $972-$1,082) | Includes drug, staff, test costs, and overheads. |  |

NGO: Non-governmental organisation. MSM: men who have sex with men. APH: Alliance for Public Health. HCV: Hepatitis C virus. HBV: Hepatitis B virus.

**Supplementary table 5:** Mean incremental costs, DALYs and incremental cost-effectiveness ratio over 2016-2030 for the status quo compared to a counterfactual scenario where there are no NGOs over 2016-2020 but they resume thereafter.

|  | **Status quo** | **No NGOs 2016-2020** | **Incremental (status quo versus no NGO 2016-20 scenario)** |
| --- | --- | --- | --- |
| **Overall cost (US$)** | **$35,600,695** | **$26,647,922** | **$8.952,773** |
| ART cost | $14,255,863 | $12,661,237 | $1,594,626 |
| NGOs cost | $21,344,832 | $13,986,685 | $7,358,147 |
| **DALYs averted** |  |  | **14,918** |
| **Mean ICER (US$)** |  |  | **$600.15** |

DALYS: Disability adjusted life years. NGOs: Non-governmental organisations. USD: US dollars. ICER: Incremental cost-effectiveness ratio.

**Supplementary figure 6:** Probabilistic sensitivity analysis of the cost-effectiveness acceptability curve of the status quo scenario vs no non-governmental organisations for 2016-2020. Blue line is half of Ukraine’s gross domestic product per capita (GDP) ($1548), and the red line is Ukraine’s GDP ($3096).

DALY: Disability adjusted life year.

**References**

1. Alliance for Public Health. Publications 2020 [Available from: <http://aph.org.ua/en/resources/publications/>.

2. Johnson WD, O'Leary A, Flores SA. Per-partner condom effectiveness against HIV for men who have sex with men. Aids. 2018;32(11):1499-505.

3. US Centers for Disease Control. Chapter 14: Reproductive, Maternal and Child Health in Eastern Europe and Eurasia: A Comparative Report2003.

4. May MT, Ingle SM, Costagliola D, Justice AC, de Wolf F, Cavassini M, et al. Cohort Profile: Antiretroviral Therapy Cohort Collaboration (ART-CC). Int J Epidemiol. 2014;43(3):691-702.

5. United Nations Department of Economic and Social Affairs. Population Dynamics 2020 [Available from: <https://population.un.org/wpp/>.

6. Morgan D, Mahe C, Mayanja B, Okongo JM, Lubega R, Whitworth JAG. HIV-1 infection in rural Africa: is there a difference in median time to AIDS and survival compared with that in industrialized countries? Aids. 2002;16(4):597-603.

7. Hollingsworth TD, Anderson RM, Fraser C. HIV-1 transmission, by stage of infection. J Infect Dis. 2008;198(5):687-93.

8. Sucharitakul K, Boily MC, Dimitrov D, Mitchell KM. Influence of model assumptions about HIV disease progression after initiating or stopping treatment on estimates of infections and deaths averted by scaling up antiretroviral therapy. Plos One. 2018;13(3).

9. Cori A, Ayles H, Beyers N, Schaap A, Floyd S, Sabapathy K, et al. HPTN 071 (PopART): A Cluster-Randomized Trial of the Population Impact of an HIV Combination Prevention Intervention Including Universal Testing and Treatment: Mathematical Model. Plos One. 2014;9(1).

10. Berenguer J, Parrondo J, Landovitz RJ. Mathematical modeling of HIV-1 transmission risk from condomless anal intercourse in HIV-infected MSM by the type of initial ART. Plos One. 2019;14(7).

11. Alliance for Public Health. Estimation of the Size of Populations Most-at-Risk for HIV Infection in Ukraine. 2018.

12. Salomon JA, Haagsma JA, Davis A, de Noordhout CM, Polinder S, Havelaar AH, et al. Disability weights for the Global Burden of Disease 2013 study. Lancet Glob Health. 2015;3(11):E712-E23.

13. Trickey A, Stone J, Semchuk N, Saliuk T, Sazonova I, Varetska O, et al. Is contact between men who have sex with men and non-governmental organisations providing harm reduction associated with improved HIV outcomes? Hiv Med. 2020.

14. Rates.org.uk E. Ukraine Hryvnia to US Dollar Spot Exchange Rates for 2019 2020 [Available from: <https://www.exchangerates.org.uk/UAH-USD-spot-exchange-rates-history-2019.html>.

15. World Bank. Consumer Price Index - Ukraine 2020 [Available from: <https://data.worldbank.org/indicator/FP.CPI.TOTL?locations=UA>.

16. Optima. Resource optimization to maximize the HIV response in Eastern Europe and Central Asia. 2020.

17. The USAID HIV Reform in Action Project. HIV Investment case study for Ukraine: Evaluation of program costs, service quality, and resource allocation for HIV expenditure in 2015. Kyiv: HIVRiA; 2018.

18. Deloitte, Latypov A, Dierst-Davies R, Sereda Y, Kerr CC, Duda M, et al. HIV investment case study for Ukraine: Evaluation of program costs, service quality, and resource allocation for HIV expenditure in 2015. 2018.
